# Concentration of Fluoride in Saliva After Fluoride Gel Application: A Randomised Clinical Trial

**DOI:** 10.1016/j.identj.2024.01.005

**Published:** 2024-05-10

**Authors:** Anna Turska-Szybka, Zuzanna Piotrkowicz, Maria Prokopczyk, Dorota Olczak-Kowalczyk, Maciej Sierakowski, Dariusz Gozdowski, Jacek Tomczyk

**Affiliations:** a Department of Paediatric Dentistry, Medical University of Warsaw, Poland; b Students’ Research Group at the Department of Paediatric Dentistry, Medical University of Warsaw, Poland; c Institute of Biological Science, The Cardinal Stefan Wyszynski University, Warsaw, Poland; d Department of Biometry, Warsaw University of Life Science, Warsaw, Poland

**Keywords:** Fluoride ions, Saliva, Fluoride gel, Randomised clinical trial

## Abstract

**Objective:**

The aim of this randomised clinical trial (RCT) was to assess the concentration of fluoride ions in saliva after applying fluoride gel and to examine the extent to which rinsing the mouth with water affects the content of fluoride ions in saliva as opposed to no rinsing after brushing teeth with gel.

**Methods:**

This single-blind RCT was conducted amongst a group of students (N = 103) and consisted of a questionnaire, clinical examination, saliva collection, and laboratory analysis. After saliva samples were collected, the students brushed their teeth for 2 minutes with Elmex Gelée (Colgate-Palmolive). Next, saliva was collected after 15 and 60 minutes from Group A (spitting out saliva after brushing) and from Group B (spitting out and rinsing with water after brushing). Statistical analysis was performed using a *t* test to compare the means between the groups.

**Results:**

Fifteen minutes after brushing, the level of fluoride increased in Group A about 15-fold to 15.33 ± 14.73 ppm and in Group B about 6-fold to 6.19 ± 5.97 ppm (*P* < .001). After 60 minutes, in Group A it decreased to 1.68 ± 0.83 ppm and in Group B to 0.60 ± 0.37 ppm (*P* < .001).

**Conclusions:**

The use of fluoride gel in the absence of mouth rinsing produced significantly higher level and a concentration of fluoride in saliva. A post-gel brushing without rinsing might be suitable for patients at high risk for caries. Hence fruther research on the effect of a mouth rinse after use of fluoride gels is eesential to confirm our findings, and to promulgate evidence-based recommendations.

## Introduction

In addition to a proper diet and hygienic behaviour, the exogenous use of fluoride is a key element in caries prevention.^1^, ^2^, ^3^, ^4^, ^5^ Fluoride gels available on the market include gels of neutral pH 2% sodium fluoride (NaF) containing 9000 ppm fluoride and low pH (acidulated phosphate fluoride [APF]) gels containing 1.23% fluoride (12,300 ppm fluoride).^5^^,^^6^ In a dental office, fluoride gel is generally applied for 1 to 4 minutes. Home use follows instructions provided in the package insert or as instructed by a dentist or a physician.

In an acidic environment, the enamel absorbs fluoride ions better, and the effectiveness of APF gel against caries has been confirmed.^5^, ^6^, ^7^, ^8^

Preparations with a high concentration of fluoride prevent the development of caries, and fluoride remaining in the saliva after application has a cariostatic effect.^9^ It has also been proven that fluoride gels inhibit erosion caused by gastroesophageal reflux.^10^ According to recommendations, fluoride gels can be used from the age of 6 years^1^, ^2^, ^3^, ^4^, ^5^^,^^11^ or 10 years.^12^

The level of fluoride in the oral cavity after application of the fluoride preparation and its clearance in saliva depends on the patient's individual factors (tongue and cheek movements, volume and rate of salivation, age, stimulants, swallowing) and variables such as type of fluoride carrier, time and method of application, and posttreatment recommendations.^13^^,^^14^ After application, fluoride is also distributed in the oral cavity and delivered to dental plaque, enamel, and soft tissues.^15^

Scientific evidence indicates that to maintain the concentration of fluoride sufficient to control the development of dental caries, the most rational methods would be those with high frequency and low concentration (eg, fluoridated toothpaste and water). In communities with access to fluoridated water, the use of professional topical fluorides has been restricted to individuals who are at high risk for dental caries, in the presence of white spot lesions. In this group of patients, enhanced prevention is recommended (eg, gels, foams, varnishes). However, in areas where there is no water fluoridation or suboptimal fluoride concentration and where there is limited use of fluoride toothpaste, the regular professional topical application may be considered.

The issue of exposure to fluoride compounds and the comparative assessment of fluoride retention in saliva after exogenous fluoridation treatments have been the subject of many studies.^7^^,^^16^, ^17^, ^18^, ^19^, ^20^ However, the number of studies evaluating the extent to which rinsing the mouth with water affects the level of fluoride ions in saliva is limited.^18^^,^^20^ Most of these studies have shown that salivary fluoride levels decrease soon after a single application.

In order to choose the most appropriate method of fluoridation for a given patient, the physician needs to know the degree of effectiveness of the method in terms of increasing the concentration of fluoride in the saliva. As described in the leaflet regarding the use of amine fluoride (AmF) gel, the gel should be spat out and the mouth rinsed after brushing, which may further reduce fluoride retention.

Elmex Gelée's (Colgate-Palmolive) leaflet contains the following information: “For the prevention and treatment of early caries in children over 6 years of age and adults: apply once a week, it is recommended to use before bedtime. Apply 1 cm of gel to the brush (about 0.5 g, ie, 6.25 mg fluoride), brush your teeth for 2 to 3 minutes. Spit out and rinse your mouth.”

The primary source of information on the application of products containing fluoride for dental practitioners and patients is the leaflet provided and the manufacturer's instruction. However, scientific institutions and societies actually do not endorse rinsing after professional application of fluoride, despite manufacturer's claims.^4^ The leaflet information encouraged the authors to investigate the validity of rinsing the mouth after brushing with fluoride gel.

The aim of the randomised controlled clinical study was to assess the concentration of fluoride ions in saliva after the use of fluoride gel and to examine the extent to which rinsing the mouth with water affects the level of fluoride ions in saliva compared to no rinsing after gel brushing. The null hypothesis was that there was difference in the saliva fluoride concentration following rinsing the mouth with water after gel brushing as opposed to no rinsing after procedure.

## Methods

The randomised clinical trial (RCT) included students of the Faculty of Medicine and Dentistry, Medical University of Warsaw (Poland), who were recruited for the study and voluntarily participated. The study consisted of 4 parts: (1) qualifying questionnaire, (2) clinical examination, (3) triple saliva collection, and (4) laboratory analysis of the obtained saliva samples. Signed and informed written consent for the study was obtained from potential participants.

### Eligibility survey

The following criteria were taken into account in the anonymous survey: age >18 years, general good health including oral health (patients without dental caries, developmental defects or erosive lesions, soft tissue diseases, salivary gland diseases, and salivation disorders), not taking antibiotics for the past 2 months, not using fluoride compounds, and no braces. In an attempt to optimise compliance, participants were advised to refrain from using topical fluorides and to refrain from consuming fluoride-rich foods and beverages to prevent confounding of study results. Exclusion criteria included pregnancy, lactation, xerostomia, periodontitis, disruption of the oral mucosa, fluorosis and other dental malformations, past fluoride overdose, allergies and systemic diseases, and hypersensitivity to the ingredients of the preparation.

### Study design

#### Randomisation

This study used a single-blind randomised design. Computer-generated randomisation was performed with the aid of a software through permuted blocks by a biostatistician (DG). Group allocation was concealed in sealed, opaque, labelled envelopes that remained unopened until the start of baseline examination.

The participants were randomised into 2 groups: Group A–APF gel for 2 minutes followed by spitting out after brushing after 15 and 60 minutes and Group B–APF gel for 2 minutes followed by spitting out and rinsing with water after brushing after 15 and 60 minutes.

#### Clinical trial procedure

Testing was performed in the morning, before breakfast or 2 hours after a meal in order to maintain a time lag before sampling and to avoid any influence of food intake on the composition of saliva as well as to minimise the influence of circadian variability. Participants did not brush their teeth on the morning of the study.

The following interventions were conducted:

1. Unstimulated mixed saliva was collected for 15 minutes into 50 mL Falcon tubes to assess the baseline fluoride concentration in the oral fluids before brushing with a gel.
2. Then participants brushed their teeth with Elmex Gelée (Colgate-Palmolive) for 2 minutes. Elmex Gelée has a fluoride concentration of 12.500 ppm (12.5 mg per 1 g gel). One gram of gel contains 33.19 mg AmF—30.32 mg Olaflur aminofluoride and 2.87 mg Dectaflur aminofluoride—and 22.1 mg NaF, 10,000 ppm NaF + 2,500 ppm AmF. The length of the applied gel strip was 1 cm (0.6 g). Participants were instructed not to swallow the gel.
3. After brushing with gel, the participants in Group A spat out excess saliva and participants in Group B spat out excess saliva and rinsed their mouth with 50 mL of tap water, following Elmex Gelée recommendations in the leaflet. Rinsing water for the patients in Group B was taken from the same source. The concentration of fluoride in drinking water in Warsaw that was used for mouthrinsing in the study was below 0.15 mg/L.
4. Fifteen minutes after brushing, participants in both groups spat out saliva for 30 seconds.
5. After 60 minutes, unstimulated saliva samples were again collected from each participants.

**Fig. 1:**
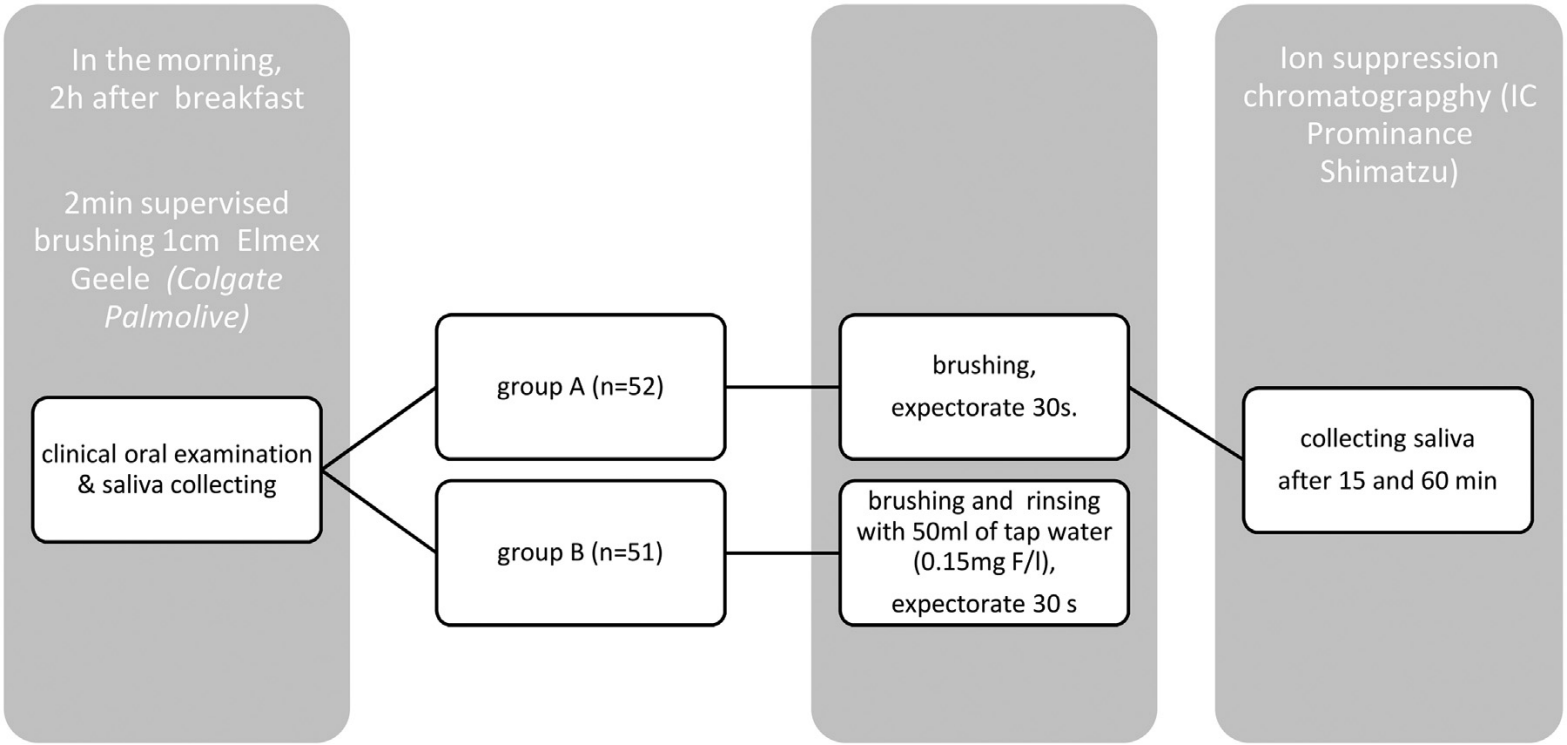
Scheme of saliva collection.

All samples for analysis were collected in the dental office by the researchers (ZP, MB) under the supervision of a dentist (ATS) (Figure 1).

### Fluorine determination

The determination of fluorine was carried out by ion chromatography with ion suppression (Prominence IC; Shimadzu); it is one of the most effective methods for the determination of hardly excited elements, such as fluorine or chlorine. In the first stage, 1.5 mL of saliva was measured into the test tube and 4.5 mL of LC-MS LiChrosolv (Merck) acetonitrile was added to deproteinise the specimen. The sample was then centrifuged at 4500 rpm for 15 minutes. The clear supernatant was transferred to chromatographic vessels. Then, 1000 µL of sample was injected. The analysis was carried out at a flow rate of 0.8 mL/min and an oven temperature of 45 °C for 45 minutes; the mobile phase was a 3.5 mM Na_2_CO_3_ solution. Milli-Q Purity Class I (Merck) deionised water was used for this procedure. The calibration curve was made on an anion standard for ion chromatography 100 mg/L F−. The study was performed at the Laboratory of Toxicology and Environmental Technologies, The Cardinal Stefan Wyszyński University in Warsaw, Poland.

### Statistical analysis

The obtained data were entered into the study form and subjected to statistical analysis using a *t* test to compare the means between the groups.

The sample size determination was based on the previous studies, for example, results of Opydo-Szymaczek and Opydo.^16^ An assumption was made that expected fluoride concentrations in saliva [mg F/L] in the 2 studied groups 15 minutes after brushing with a gel would be, respectively, about 15 in the control group and 30 in the test group (expected difference, 15) and standard deviation (SD) would be about 26.5, whilst 60 minutes after brushing with a gel the mean fluoride concentration would be respectively 2.8 vs 4.0 (expected difference, 1.2) and SD would be about 2.1. The sample size determination was performed assuming power of 80% and confidence level of 95% (*α* = 0.05, *β* = 0.20) for the *t* test. Based on such assumptions, the required sample size for each group equalled about 50 observations.

Statistica 13 (StatSoft) software was used for the analysis, and the assumed significance level was *P* < .05. The RCT was conducted in accordance with ethical standards and the Declaration of the World Medical Association of Helsinki (1964, final version of 2008). Approval of the Bioethics Committee of the Medical University of Warsaw No. KB/224/2017 was obtained.

## Results

**Fig 2:**
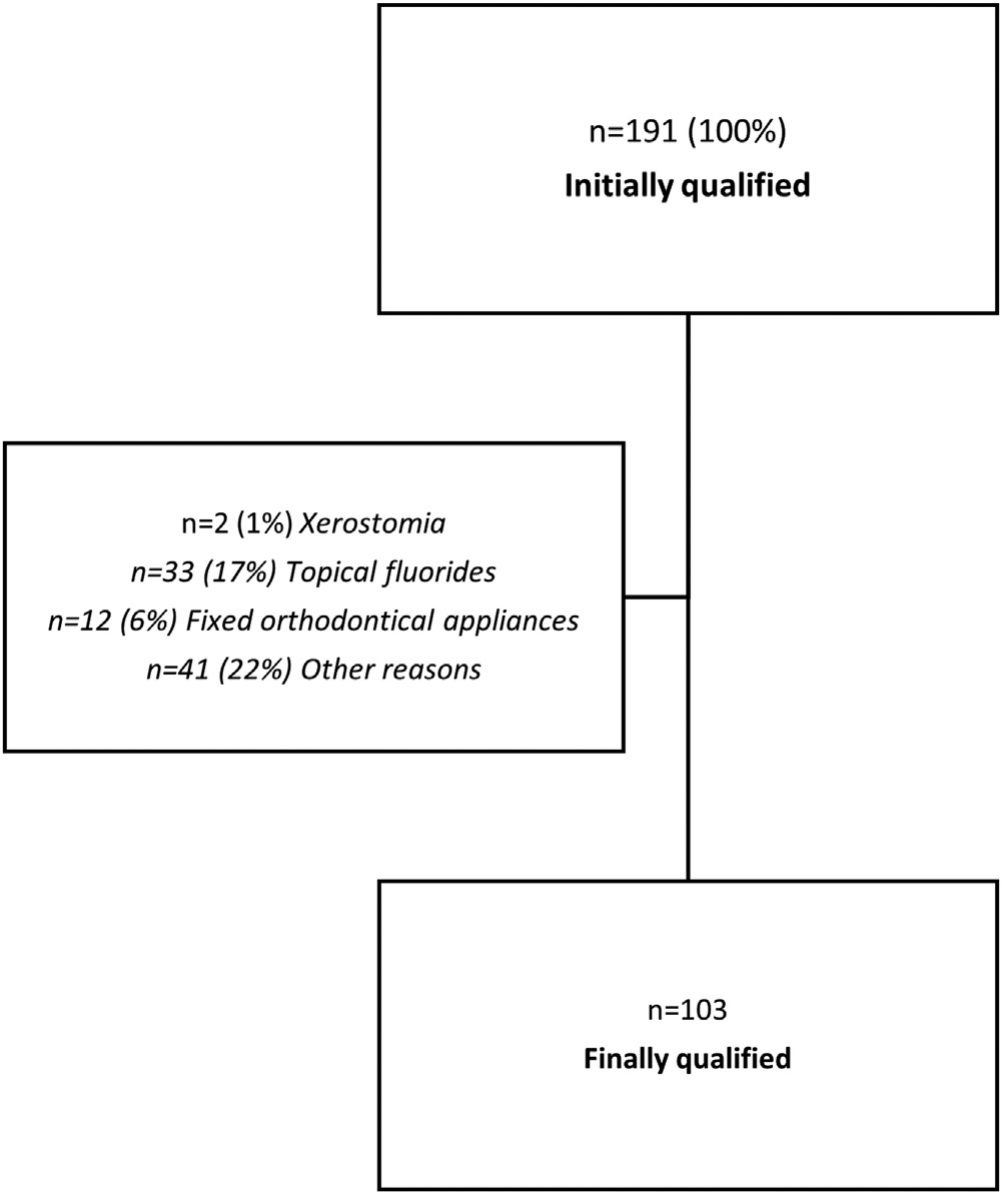
Selection of participants for research.

A total of 191 students aged 21 to 25 years participated in the randomised clinical study, of whom 103 participants met the inclusion criteria (Figure 2).

Fluoride concentrations in saliva before and after gel brushing are presented in the Table. Group A included 52 participants who spat saliva into test tubes after brushing. Group B included 51 participants who rinsed their mouth with water after using the gel. Baseline salivary fluoride concentration in both study groups was quite similar (*P* > .05).

**Table:** Fluoride concentrations in saliva [mg F/L].

Type of examination	Group A (n = 52)			Group B (n = 51)			*P*
	Min	Max	Mean	Min	Max	Mean	
Fluoride concentration before brushing with a gel	0.00	1.920	0.19 ± 0.38	0.00	0.328	0.08 ± 0.10	.075
Fluoride concentration 15 min after brushing with a gel (study 1)	0.212	86.172	15.33 ± 14.73	0.20	22.852	6.19 ± 5.97	<.001*
Fluoride concentration 60 min after brushing with a gel (study 2)	0.012	6.475	1.68 ± 0.83	0.00	0.100	0.60 ± 0.37	<.001*

⁎ Statistically significant *P* < .05.

Fifteen minutes after brushing (study 1), the fluoride level in Group A increased about 15-fold, reaching an average concentration of 15.33 ± 14.73 ppm. In Group B, the average concentration increased only about 6 times to the 6.19 ± 5.97 ppm. The fluoride concentration in the saliva of Group A was almost 2.5-fold higher than that of Group B, and the difference was statistically significant. Sixty minutes after brushing (study 2), the level of fluoride in Group A decreased to 1.68 ± 0.83 ppm on average and in Group B to 0.60 ± 0.37 ppm. In this case, there was also a difference at the level of statistical significance.

## Discussion

Fluoride retention in the oral cavity after topical fluoride application is considered an important factor in the clinical efficacy of fluoride, which depends on its concentration and the method of administration. Low concentrations of fluoride in saliva are sufficient to inhibit demineralisation and enhance remineralisation. Topical fluoride balances the cycle of demineralisation and remineralisation in saliva by increasing fluoride uptake during demineralisation and increasing remineralisation. In this process, saliva acts as a fluoride reservoir and exerts both local and systemic effects.^5^ The constant presence of fluoride in saliva helps to replenish minerals lost from the tooth surface due to changes in oral pH.^21^ It should be noted that the time factor plays an important role in the bioavailability of fluoride after topical application.

Many studies have dealt with the effect of rinsing the mouth with water on fluoride levels after using toothpaste.^17^^,^^19^^,^^20^^,^^22^, ^23^, ^24^, ^25^ However, there are few studies on the effect of rinsing the mouth with water on the level of fluoride after using gels, which have significantly higher fluoride concentrations than toothpastes.^26^, ^27^, ^28^, ^29^, ^30^, ^31^, ^32^ Elmex Gelée's (Colgate-Palmolive) leaflet contains the following recommendation after gel brushing for 2 to 3 minutes: “Spit out and rinse your mouth.” This information encouraged the authors to investigate the validity of rinsing after brushing with fluoride gel.

The findings of the present randomised clinical study indicate that the null hypothesis has been confirmed. A significant increase in salivary fluoride levels after brushing was observed in participants who did not rinse their mouth with water (15.33 ± 14.73 vs 6.19 ± 5.97). It should be emphasised that the study group was homogeneous in terms of age and area of residence, which increases the credibility of the obtained research results. Given the systematic underestimation of fluoride concentrations, the ion assessment with the method used in this study represents the true bioavailability at the time of saliva sampling.

Comparison of fluoride levels in saliva at baseline showed differences between both groups, although they were not statistically significant. In an attempt to optimise compliance, researchers advised participants to refrain from using topical fluorides and to refrain from consuming fluoride-rich foods and beverages to prevent confounding of study results. The students did receive instructions regarding their diet and the last oral hygiene procedure. The participants with general good health, including oral health (patients without dental caries, developmental defects or erosive lesions, soft tissue diseases, salivary gland diseases, or salivation disorders), not taking antibiotics for the past 2 months, not using topical fluorides, and with no braces were included. Although saliva flow was not examined, patients with xerostomia or salivary gland diseases were excluded. Considering all the abovementioned factors, no significant differences between groups were expected. In this group of students of the same age—despite the uniform protocol—individual saliva secretion, postbrushing rinsing behaviour in the evening before examination, individual characteristics of saliva, and consumption of food and beverages may have had an influence on a difference in the fluoride concentration before brushing with the gel between Group A and Group B. Thus, most of the abovementioned factors were similar for all the participants in this randomised clinical study, though the differences in salivary kinetics remained specific to each participant. It is worth mentioning that the difference in fluoride concentration before brushing with the gel between Group A and Group B was not statistically significant. Thanks to protocol-based randomised study design—which represents the gold standard of medical evidence—and inclusion and exclusion criteria, risk of bias at baseline was avoided.

Covalently bonded fluorine in AmF does not dissociate in the same way as ionic fluorine from NaF. AmF in fluoride gels does not interact with other ions present in solution, whilst the release of F− ions from NaF changes when other ions dissolve in the same solution. For the purpose of fluoride delivery, aminofluorides are more stable compounds and can deliver F ions more efficiently to prevent caries.^33^ The inclusion of the gelling agent, sodium carboxymethyl cellulose, in the APF gel ensures the viscosity of the preparation, facilitating gel penetration.^34^ There have been suggestions of fluoride ion toxicity to oral cariogenic bacteria such as *S mutans* when topical fluoride is used in high concentrations, and this effect may persist for extended periods of time.

APF gel is considered clinically effective in reducing caries. Routine use of professionally applied fluoride gel provides benefit to persons at high risk for tooth decay, especially those who do not live in areas with fluoridated water or brush their teeth daily with fluoride toothpaste.^7^ The widely used APF gel is acidic and ionic.^35^ Thus, the chemical reaction of APF gel with enamel produces a quick deposition on the tooth surface.^35^, ^36^, ^37^

Results similar to ours were obtained by Stookey et al,^26^ who proposed refraining from rinsing after topical application of fluoride in order to obtain maximum local fluoride efficacy. Another study showed that an average of 1.8 mg F remained in the mouth after brushing with 0.5 g fluoride gel containing 1.23% acidified phosphate fluoride.^27^ Since the total oral surface area is greater in adults than in children, the total amount of fluoride absorbed into oral tissues will also be relatively greater in adults. Research by Fatemeh et al^31^ demonstrated that instantaneous rinsing with water after applying the APF gel reduced the effect of the preparation on the acidity of dental plaque. By contrast, this effect was not observed in the case of rinsing for 15 minutes after application of APF gel and with the use of casein phosphopeptide–amorphous calcium phosphate after fluoridation with APF gel and subsequent rinsing of the mouth.^32^ Also, the study by Jabin et al^38^ showed a statistically significantly higher fluoride concentration in saliva for the group of patients after application of APF without rinsing the mouth after 5 minutes and 1 hour (4.79 ± 0.746 and 4.01 ± 0.940, respectively) vs baseline (1.94 ± 0.548).

Contrary to the aforementioned studies, Borysewicz-Lewicka et al,^29^ comparing the effect of rinsing and its absence on the level of fluoride after gel brushing, showed that after brushing with Elmex Gelée, 0.7 to 1.8 mg of fluoride (on average, 1.2 mg) remained in the mouth in 7-year-old patients and 0.5 to 1.8 mg F (mean, 1.3 mg) in 11-year-old patients (*P* > .05). Similar, although slightly higher, values were obtained by Heath et al^27^ in adults (on average, 1.8 mg). The results of Delbem et al^28^ showed a higher level of fluoride in the saliva after the application of the APF gel than before the application, but the differences between the rinsing and the nonrinsing groups of patients were statistically insignificant. In another study, Delbem et al^30^ concluded that rinsing with water after topical application of the fluoride preparation did not adversely affect the absorption of fluoride ions and the formation of calcium fluoride on the enamel surface, regardless of the method of application. However, the inconsistency of the results of the cited studies may be due to the size of the research sample or different research methodology.

The kinetics of fluoride in mouth fluids is well understood. The physiologic fluoride concentration in saliva is approximately 0.02 ppm F, which can be significantly increased by topical application of a fluoride, and returns to baseline levels in saliva after more than 30 minutes.^39^ Consistent with the biphasic pattern of clearance, there is an initial rapid increase with a peak at 15 minutes followed by a decline at 45 minutes. In the present study, there was a significant decrease in fluoride levels after 60 minutes. Nuca et al^40^ observed that after a peak increase of fluoride in saliva, the concentration of fluoride slowly decreased. Naumova et al^14^ found a peak increase in fluoride concentration in saliva immediately after brushing and persisting for at least 30 minutes. Several authors have conducted studies that used similar time intervals. Longer observation intervals (2, 12, 24, and 48 hours) from the moment of initial fluoridation would allow further monitoring of fluoride concentration in saliva, but this requires further research.

The bioavailability of F− immediately after tooth brushing and clearance are strongly related to the individual salivary flow rate and are also not constant throughout different areas of the oral cavity. In the present study, the rate of salivation in students was not assessed, which may be a limitation of the study and may be a confounding factor, creating a risk of bias.

Retention of fluoride in saliva after using concentrated gels is extremely important in patients at high risk for caries and in patients during and after radiotherapy, especially amongst those who do not live in areas with fluoridated water or do not brush their teeth daily with fluoride toothpaste. The European Academy of Pediatric Dentistry, the American Dental Association, and World Dental Federation FDI promoting oral health through fluoride have similar recommendations regarding topical fluoride applications based on caries-risk assessment. Community water fluoridation is seen by these organisations as an important element in a complex approach to caries prevention, to which the use of topical fluoride in relation to caries-risk assessment is added.^8^^,^^41^ Topical fluoride has made a contribution not only to prevent the onset of carious lesions but also to arrest them at early stages.^42^

## Conclusions

Studies have shown that fluoride levels in saliva after using a high-fluoride gel are higher than physiologic levels. Significantly higher concentration of fluoride in saliva occurs after brushing teeth with fluoride gel without subsequent rinsing of the oral cavity. The results of the randomised clinical study show that the use of a fluoride gel followed by spitting out the excess gel without rinsing the mouth promotes greater retention of fluoride in saliva. A post–gel brushing without rinsing might be suitable and preferable for a group of patients at high risk for caries, especially for those who rinse extensively after brush-on gel. Demonstrating this relationship may contribute to the discussion on possible continuation of research and verification of recommendations of abstaining from rinsing the mouth after the use of gels.

## Conflict of interest

None disclosed.

## Appendix Supplementary materials

Image, application 1
