# Supplementary material for: Concentration of Fluoride in Saliva After Fluoride Gel Application: A Randomised Clinical Trial
Source: Int Dent J. 2024 May 10;74(4):794–800. doi: 10.1016/j.identj.2024.01.005 (PMC11287168; doi:10.1016/j.identj.2024.01.005)
Supplement: Supplementary file 1 [file mmc1.pdf]

RVG 06269

Ref: ELG-EU-61

ELMEX® GELÉE

Active ingredient: Amine Fluoride (Olafur, Dectaflur)
Sodium Fluoride

ATC Code: A01AA51

PERIODIC SAFETY UPDATE REPORT

VALID FOR THE FOLLOWING MAH:

ELMEX MEDICAL CARIËSPROTECTIEGEL 12,5 MG/G, TANDGEL

	NETHERLAND	GABA GMBH	RVG 06269

ELMEX MEDICAL GEL, 1,25% FLUORIDE, GEL VOOR DENTAAL GEBRUIK

	BELGIUM	GABA GMBH	1362 LC 1 F 7
ELMEX GELEE	SLOVAKIA	GABA GMBH	87/0006/82-S
ELMEX GÉL	HUNGARY	TEVA BUDAPEST	OGYI-T-1646/01-02
ELMEX GELÉE	GERMANY	GABA GMBH	6169101
ELMEX GELÉE	LUXEMBURG	GABA GMBH	0311/00056736

PERIOD COVERED BY THIS REPORT: 01.08.2009 – 31.07.2012

INTERNATIONAL BIRTH DATE
17.06.1969 (SWITZERLAND)

EU BIRTH DATE:
03.07.1969 (BELGIUM)

DATE OF REPORT: SEPTEMBER 2012

CONFIDENTIAL
MAY NOT BE USED, DIVULGED, PUBLISHED OR OTHERWISE DISCLOSED WITHOUT WRITTEN CONSENT OF [redacted]

A

Prepared by:	
Date: ………………..	Signature …………………………..
	[redacted] Associate in Pharmacovigilance, [redacted]
Reviewed by :	
Date: ………………..	Signature …………………………..
	[redacted] Qualified [redacted] Pharmacovigilance, [redacted]
Date: ………………..	Signature …………………………..
	[redacted] Deputy QPPV [redacted]

A

C

CONFIDENTIAL
MAY NOT BE USED, DIVULGED, PUBLISHED OR OTHERWISE DISCLOSED WITHOUT WRITTEN CONSENT OF
[redacted] A

CONTENTS

	SECTIONS	1st PAGE #
	Executive Summary	1
1	Introduction	3
2	Worldwide Marketing Authorisation Status	5
3	Update of Regulatory Authority or MAH Actions Taken for Safety Reasons	6
4	Changes to Reference Safety Information	7
5	Patient Exposure	8
6	Individual Case Histories	9
	6.1 General considerations	9
	6.2 Cases presented in the Principal Line Listing	9
	6.3 Overview – Principal Line Listing Summary Tabulation	10
	6.4 Analysis of individual case histories	10
	6.5 All death case	15
7	Studies	15
	7.1 Newly analyzed company-sponsored studies	15
	7.2 Targeted new safety studies	15
	7.3 Published safety studies	15
	7.4 Other studies	16
8	Other Information	16
	8.1 Lack of efficacy	16
	8.2 Late breaking information	16
	8.3 Risk-management plan	16
	8.4 Risk-benefit analysis report	16
9	Overall Safety Evaluation	16
	9.1 Cumulative Perspective: Serious Unlisted Reactions	16
	9.2 Cumulative Perspective: Serious Listed Reactions	16
	9.3 Cumulative perspective: Non-Serious Unlisted Reactions	17
	9.4 Increased Reporting Frequency of Listed Reactions	17
	9.5 Changes in Characteristics of Listed Reactions	17
	9.6 Interactions	17
	9.7 Experience with Overdose	17
	9.8 Abuse and Misuse	17
	9.9 Experience with Pregnancy and Lactation	18
	9.10 Experience in special patient groups	18
	9.11 Effects of long-term treatments	18
	9.12 Cases from non-health care professionals	18
	9.13 Prescription errors/medication errors	19
10	Conclusions	19
	**APPENDICES**	
1	Worldwide marketing authorization status	20
2	Core Company Data Sheet	23
3	Company Core Safety Information	31
4	Principle Line Listings – includes clinical trial, spontaneous, literature and regulatory reports	38
5	Principle Line Listing Summary tabulation	50
6	Cumulative Summary Tabulation of Serious Unlisted Reactions	55
7	Summary tabulation of serious listed reactions from the Principle Line Listing	58
8	Summary Tabulation of Serious and Non-serious Unlisted Reactions	60
9	Literature reference	64
	**ANNEXES**	
1	All spontaneous reports of non-serious listed reactions and tabulation	65
2	All serious and non-serious (listed and unlisted) medically unconfirmed adverse reactions and tabulation	75

EXECUTIVE SUMMARY

Elmex® gelée is fluoride toothpaste containing 33.19 mg amine fluorides (30.32 mg olaflur and 2.87 mg dectaflur) and 22.1 mg sodium fluoride per gram of gel. This corresponds to a total fluoride content of 1.25 %.

Elmex® gelée is used topically in caries prophylaxis for the fluoridation of tooth enamel. The dental gel promotes remineralization of initial caries and is suitable in the treatment of hypersensitive dental necks. In addition, the amine fluorides (olaflur/dectaflur) have antimicrobial properties.

Elmex® gelée is commercialised in several countries. In most of the countries, elmex® gelée has an OTC status. In some countries, it is authorised as a prescription medicine and in Spain as a Dentifrice.

The trade name elmex® gelée is also used in most countries with slight deviations in Finland, the Netherlands, Belgium and Austria.

This PSUR covers the period from 01 August 2009 through 31 July 2012.
During this period, there have been no suspensions or failures to grant renewal of the Marketing Authorisation, no change to the formulation of the product, no changes in the target population and no changes in the benefits of using the product. The Core Company Safety Information document has been revised in order to integrate and harmonize the information from all European SmPC as explained in section 4. The SmPC is being revised accordingly.

This report confirms that the overall safety profile of elmex® gelée is very good. With an exposure of more than [redacted] packs during the period of this PSUR, there have been 59 medically confirmed spontaneous or authority issued reports of adverse reactions from which there were five serious adverse reaction reports. This represents 1 medically confirmed report per more than [redacted]acks of the product placed on the market. A

There were five (5) serious cases reported during the PSUR period. Two were related to an accidental ingestion of the product by children, one was an allergic shock and the two others were mouth inflammation considered as serious by the reporters. From the 54 spontaneous non-serious reports, most of the cases were related to gastrointestinal disorders as could be expected for this type of product. 24 reports contained only reactions that were listed, while 30 reports contained at least 1 non-serious unlisted reaction. There was no evidence of a specific signal that could be identified from the analysis of the cases and that could lead to new relevant information changing the risk-benefit balance of the product.

From the analysis of 51 consumer reports, the medically unconfirmed cases concern essentially minor gastrointestinal disorders and more specifically minor reactions in the oral cavity, which could usually be expected with this type of product. One serious case of an allergic reaction was also reported after accidental ingestion of an unknown amount

of product by a child. That case was not medically confirmed. Overall, consumer reports did not provide any additional information in comparison to the medically confirmed reports.

Overall, the Summary of Product Characteristics contains sufficient information to inform physicians, dentists, pharmacists and patients about the occurrence of adverse drug reactions and to warrant the safe use of elmex® gelée which still has an excellent risk-benefit ratio when used under the conditions stipulated in the Summary of Product Characteristics.

1. INTRODUCTION

This report is a Periodic Safety Update (PSUR) for the stomatological agent elmex® gelée which has been commercially available for decades. This PSUR is based on the Guidance "Volume 9A of the rules governing medicinal products in the European Union – Guidelines on pharmacovigilance for medicinal products for human use, September 2008" and does not follow the new format defined in the European Medicine Agency Guideline on Good Pharmacovigilance Practices, Module VII – Periodic Safety Update Report.

Adverse events and adverse reactions are data entered, analyzed and assessed in the validated safety database [redacted] in a "state of the art" hosted environment for [redacted] includes MedDRA (Version 15.0) and WHO Drug coding. Regulatory reports, including line listings and summary tabulations are now being generated from [redacted]

A

Definitions that are used in [redacted] are based on Volume 9a. The current PSUR includes the following:

- Spontaneous reports – include unsolicited communication by HCPs or consumer to the MAH, regulatory authority or other organizations that may describe suspected AEs, if one or more medicinal products were administered and if not derived from a study or organised data collection scheme
- Medically unconfirmed reports – include reports from patients and other consumers, not medically confirmed
- Adverse reaction reports received from regulatory authorities worldwide:
  Spontaneous and non-spontaneous reports from HCPs
  Reports from Patients and other Consumers (not medically confirmed)
- Principal Line Listing – includes the following:
  - Spontaneous serious adverse reactions and non-serious unlisted reactions
  - Spontaneous non-serious unlisted drug reactions
  - Serious drug reaction reports from studies (attributable to the medicinal product by the investigator or sponsor)
  - Literature reports for serious or any non-serious unlisted drug reactions
  - Regulatory authority reports sent to the MAH for serious drug reactions
- Summary Tabulation for Principal Line Listing – includes all above categories of the Principal Line Listing by SOC and source.

Brief introduction of elmex® gelée

Active Ingredients

1g elmex® gelée contains 33.19 mg amine fluorides (30.32 mg olaflur and 2.87 mg dectaflur) and 22.1 mg sodium fluoride. This corresponds to a total fluoride content of 1.25 %.

Description of organic fluorides (amine fluorides)

Olaflur:
- CAS-No.[redacted]
- N,N`,N`-tris(2-hydroxyethyl)-N-octadecyl-1,3-propanediamine dihydrofluoride
- amine fluoride 297
- In-house monograph

Dectaflur:
- CAS-No.[redacted]
- 9-octadecenylamine hydrofluoride
- amine fluoride 335
- In-house monograph

Description of inorganic fluoride:

Sodium Fluoride:
- CAS–No.:[redacted]
- PHEUR

Excipients

Formulation 447/1716 contains:
Water, propylene glycol, flavouring agent 1304 (dl-menthone, apple aroma, banana aroma, peppermint oil and spearmint oil), hydroxyethyl cellulose, saccharin.

This formulation is valid for all market.

See also Table 1: Overview of formulations approved, page 5.

Indications

Indications include caries prophylaxis, treatment of initial caries lesion and the treatment of hypersensitive dental necks.

See also section 2. World-wide Marketing Authorisation Status.

Mechanism of action

Elmex® gelée is used topically in caries prophylaxis for the fluoridation of tooth enamel. The dental gel promotes remineralisation of initial caries and is suitable in the treatment of hypersensitive dental necks. The high affinity of the amine fluorides for the surface of the tooth results in accumulation of fluoride in the dental enamel. At the same time, the

solubility (and therefore also the demineralisation) of the enamel is reduced. In addition, the amine fluorides olaflur/dectaflur has antimicrobial properties.

Package size

Elmex® gelée is available in the package sizes of:
- 25 g (in all 17 countries, where elmex® gelée is marketed)
- 38 g (in Belgium, Germany, Luxemburg and the Netherlands)
- 215 g (in Belgium, Czech Republic, Finland, Germany, Hungary, Italy, Poland, Luxemburg, Slovak Republic and in Switzerland)

Details of the worldwide marketing authorisation status are provided in section 2.

2. WORLDWIDE MARKETING AUTHORISATION STATUS

Elmex® gelée is commercialised in several countries. According to the date of registration of elmex® gelée, the countries where the product has been placed on the market are listed in Appendix 1.

Elmex® gelée was first marketed in Switzerland on 17.06.1969 (IBD), followed by Belgium on 03.07.1969 (EBD).

In most of the countries, elmex® gelée with the pack size of 25g has an OTC status. In some countries, i.e. Finland, The Netherlands and Israel, it is authorised as a prescription medicine, and in Spain, elmex® gelée is registered as a dentifrice.

The trade name elmex® gelée is used in most of the countries. A slightly deviated name is registered in Finland (elmex® dental gel), the Netherlands (elmex® medical cariesprotectiegel 12,5 mg/g, tandgel) and Austria (elmex® Dentalgel).

The indication is differently described in the SPCs. Some indications describe the therapeutic treatment and some, the mechanism of action of the active ingredients.

Country specific differences in the composition are as follows:

Finland
Due to harmonisation purposes, the formulation 447/104, which was the first developed composition of elmex® gelée, was used until the new formulation 447/1716 was approved in September 2002.

The new formulation contains no colouring agent, no preservatives agent, and no abrasive agent and has a different aroma composition. For more details about composition of elmex® gelée, see section 3 on page 5 - 6.

The total fluoride concentration remains the same as 1.25 % fluoride.

Netherlands
elmex® gelée was available in the Netherlands with two different pharmaceutical dosages, a total fluoride content of 1.25 % (formulation 447/1716) and a total fluoride content of 0.4 % (formulation 447/1761).

The difference between these two formulations mainly was in the content of fluoride. The formulation 447/1716 contained about 85 % less sodium fluoride.

Due to harmonisation purposes, the registration of elmex® gelée 0.4 % fluoride, has been omitted by the MAH in 2003.

3. UPDATE OF REGULATORY AUTHORITY OR MAH ACTIONS TAKEN FOR SAFETY REASONS

During the period of this PSUR, there have been no suspensions or failure to grant renewal of the Marketing Authorisation for elmex® gelée, and no restrictions for distribution. There has been no change to the formulation of the product or changes in the target population.

Before the period of this PSUR, the reasons for a change of formulation from 447/402 to 447/1716 were as following:

- *Addition of Propylene Glycol*- The viscosity of the gel has been improved by the use of propylene glycol as solvent and solvent mediator (no longer any liquefaction of the final product). Due to propylene glycol the hydrolytic activity in the aqueous system of elmex® gelée is slowed down.
- *The flavouring agent*- cinnamon oil (flavour 19B) has been replaced with flavour 1304, thus removing the allergic potential and preventing burning in the mouth. The acceptance of the flavour is thereby improved.
- *Methylparabene*- Since the Olaflur/Dectaflur combination has a preservative effect on is own, Methylparabene for this purpose can be omitted. This also removes a potential cause of allergy.
- *Color C.I. 16255*- By dropping the colorant from the formula, a further allergic potential is avoided.

Table 1: Overview of formulations approved

**Excipients in Formulation 447/1716** (Third and current composition)	**Excipients in Formulation 447/402** (Second composition developed)	**Excipients in Formulation 447/104** (First composition developed)
Water<br>Flavouring agent 1304<br>dl-menthone<br>apple flavour<br>banana flavour	Water<br>Flavouring agent 19B<br>menthol nature<br>menthone synthetic<br>banana flavour	Water.<br>Flavouring agent 796<br>menthol<br>anise oil<br>peppermint oil

peppermint oil<br>spearmint oil<br><br><br>Hydroxyethyl cellulose<br>Saccharin<br>Propylene glycol	peppermint oil<br>spearmint oil<br>cinnamon oil<br><br>Hydroxyethyl cellulose<br>Saccharin<br>Methylparabene<br>Colouring agent C.I.16255	spearmint oil<br>vanillin<br><br>Saccharin<br>Methyl parahydroxybenzoate<br>(=methylparaben)<br>Sodium riboflavin phosphate<br>Sodium metaphosphate<br>Guar gum

4. CHANGES TO REFERENCE SAFETY INFORMATION

As the official local data sheets are slightly different from each other and a harmonisation of the safety information is considered, the first Core Company Data Sheet (CCDS, version 1), has been prepared on the basis of the German official safety information and on the basis of all ADRs reported in the PSUR. Within the regular internal update of the PSUR, the CCDS has been reviewed by changing the ATC code only. The new ATC code A01AA51 for sodium fluoride and combination, which is more suitable for elmex® gelée (former ATC code A01AA30 for combination of fluoride) has been introduced in version 2 of the CCDS.

The CCDS, version 3, has been changed in the format while in 2009, the following sentence has been added in version 4 of the CCDS, under the section 'Posology and method of administration – Use at home': "The total time of application (brushing and residence time) must not exceed 5 minutes." Furthermore, additional paragraphs have been introduced to the CCDS and CCSI for the use of elmex® gel in group prophylaxis

With respect to the Company Core Safety Information (CCSI), which has been prepared for determining listed and unlisted ADR reports, it has been progressively revised to reflect the updates of the CCDS and is now available in version 5.

The German SmPC was chosen as reference SmPC for other countries.

In new EU Member States such as Slovak Republic, Czech Republic and Hungary, the dossiers of all pharmaceutical products had to be updated due to EU accession and the SPCs for elmex® gelée were harmonised with the German SPC in 2003.

The Belgian SmPC had been adapted according to the Belgian Template for SmPC and safety information had been harmonised with the German SPC in September 2004. In 2004 the harmonisation of the Dutch SmPC has been submitted with approval in April 2006.

The harmonisation of the Finnish SmPC was submitted in 2005 and approved in 2006.

In order to harmonize the format of the CCSI for all company products and integrate the minor differences between the local SmPCs into the Core Company Information, the CCSI has been further revised during the period of this PSUR.

The Core Company Data Sheet is presented in Appendix 2 and the revised Core Company Safety Information (version 7) in Appendix 3. As this PSUR is submitted to different countries, the local SmPC will be attached to each submission.

5. PATIENT EXPOSURE

In this PSUR, the number of the packs sold on the markets during the period of 1 August 2009 to 31 July 2012 has been used as a comparative factor for estimating the frequency of patient exposure since it is impossible to estimate the patient exposure and the daily dose for elmex® gelée. Reasons therefore are:
Elmex® gelée has two modes of application

- a) tooth brushing with 1-2 cm gel (approximately 0.5 g gel) per week recommended at home or several times per month or year in group prophylaxis (e.g. school) and
- b) tray application; the dosage depends on the tray size (in general 3-8 g gel)

In total more than [redacted] of elmex® gelée have been sold between August 2009 and July 2012. More details are provided in table 2.

Table 2: Number of sold packs

Country	Pack size	Total packs sold Aug-Dec 2009*	Total packs sold 2010	Total packs sold 2011	Total packs sold Jan-Jul 2012	Total packs sold Aug 2009- Jul 2012
Germany	25g, 38g, 215g	[redacted]	[redacted]	[redacted]	[redacted]	[redacted]
Belgium	25g,215g	[redacted]	[redacted]	[redacted]	[redacted]	[redacted]
Slovakia	25g,215g	[redacted]	[redacted]	[redacted]	[redacted]	[redacted]
Netherland	25g,215g	[redacted]	[redacted]	[redacted]	[redacted]	[redacted]
Hungary	25g,215g	[redacted]	[redacted]	[redacted]	[redacted]	[redacted]
Other countries**	25g, 38g, 215g	[redacted]	[redacted]	[redacted]	[redacted]	[redacted]
Total per year		[redacted]	[redacted]	[redacted]	[redacted]	[redacted]
Total packs sold between August 2009 and July 2012 = [redacted]						

* 2009 Aug-Dec estimated as 5/12 of year 2009.
** Other countries include: Switzerland, Italy, Finland, Czech Republic, Finland, Austria, Poland, Portugal, Croatia, Slovenia, Israel, South Africa and Spain.

6. INDIVIDUAL CASE HISTORIES

6.1 General Considerations

All individual cases reports meeting the criteria defined below during the review period are presented in the Principal Line Listings (see Appendix 4).

- All serious adverse reactions and non-serious unlisted adverse reactions from medically confirmed spontaneous reporting;
- All serious adverse reactions (attributable to the medicinal product by either investigator or sponsor) available from post-authorisation safety studies (PASS) and other studies or named-patient/compassionate use:
- All serious adverse reactions, and non-serious unlisted adverse reactions, from literature.
- All serious adverse reactions transmitted to the Marketing Authorisation Holder by worldwide regulatory authorities.

In addition, the types of cases referenced below are included as line-listings in the form of an annex to the PSUR:

- All non-serious listed adverse reactions from non-serious listed spontaneous reports (Annex 1)
- All serious and non-serious (listed and unlisted) adverse reactions reported by Patients/Consumers and other non-healthcare professionals (not medically confirmed). (Annex 2)

Adverse events are assessed for:

- Seriousness
- Causality
- Whether listed or unlisted

The definition of a serious event is one that is fatal, life threatening, results in significant disability or incapacity, results in hospitalisation or prolongs hospitalisation, causes a congenital anomaly/birth defect, is another significant event (i.e. events judged to be medically serious or which are significant by specification in certain trials) or results from a suspected transmission of an infectious agent via the medicinal product. An unlisted event is one whose nature, severity, specificity or outcome is not consistent with the information included in the CCSI.

6.2 Cases Presented in the Principal Line Listing

The Principal Line Listing includes Spontaneous, clinical trial, literature and Regulatory Authority Reports as previously defined.

To summarize briefly, there were five serious medically confirmed ADR during the period covered by this PSUR. There were 30 non-serious unlisted medically confirmed

spontaneous reports. There were no reports from literature searches relevant to elmex® gelée and no reports from clinical studies.

6.3 Overview – Principal Line Listing Summary tabulations

The Principal Line Listing Summary Tabulation is included as Appendix 5 and presented by SOC and source.

6.4 Analysis of Individual Case Histories

In this chapter, spontaneously reported cases relevant to the safety assessment of the product are discussed according to the most prominent condition, i.e. in descending order: serious unlisted, serious listed, non-serious unlisted, non-serious listed.

6.4.1 Serious Unlisted Reports

Four medically confirmed serious unlisted reports were spontaneous notified to the company.

Among the four spontaneous cases, two are related to an accidental ingestion by children as mentioned below:

Case ID	Source	Product
[redacted]	Spontaneous	elmex® gelée

B

Initial information was received, in [redacted] on 08Sep2009, from the father of a female infant. On the morning of 05Sep2009 this 21 month old infant swallowed an unknown amount of elmex Fluoride Therapy Gel and experienced a failure to respond to any attention, anamnestic symptoms, contraction of the pupils, vomiting, nausea, closing of the eyes/drowsiness, and crying. The child, who weight 12 kg, had opened the safety cap and ingested the toothpaste before she had eaten. Follow up with the father on 08Sep2009 revealed that she had actually consumed between nine to eleven grams of the toothpaste. Approximately one hour after ingestion, she vomited and then cried for 20 minutes. Her parents tried to give her milk, but she could only drink a few sips because she failed to respond normally. Subsequently, she became nauseous, her pupils contracted, her eyes closed, she became drowsy, and she stopped responding. The parents contacted a poison control center and were advised to take her to a hospital. Follow up with the head physician of the emergency room on 10Sep2009 indicated that she was anamnestic upon arrival. She was observed for four hours, but was not admitted to the hospital and no treatment was given. The physician was unable to confirm whether or not she had experienced intoxication due to the toothpaste. The consumer had fully recovered prior to discharge from the hospital

Case ID	Source	Product
[redacted]	Spontaneous	elmex® gelée

B

Initial information was received, in [redacted] on 10Dec2010, along with additional information received on 13Dec2010, from the dentist of a female consumer. This 11 year old consumer, with relevant history of streptococcus mutans and lactobacillus in her oral

flora, used elmex® gelée and accidentally ingested some of the product. She experienced a severe allergic reaction (originally thought to be anaphylactic shock), erosion of the mucosa at the base of her tongue and gingiva, mucosal irritation, an elevated temperature, her body felt cold as ice, she had loss of taste, discomfort in swallowing, she was shivering and crying. Her mother first applied the product via a dental splint on 09Dec2010, but a bigger amount than what was recommended was used. The dental split was left in her mouth for approximately one hour and directly after removal she had loss of taste and discomfort in swallowing. The dentist noted that it could not be excluded that a certain amount of the product was accidentally swallowed. Approximately one and a half to two hours after removal of the product, she experienced shivering, was crying and her body felt as cold as ice. She was taken to the emergency room on 09Dec2010, where she took calcium tablets and it was recommended that use of the product be discontinued. She also drank milk and water to improve the swallowing disturbance. On 10Dec2010, she was seen by the dentist and her mother reported elevated temperature and shivering in the morning, but these events had resolved by the time of her afternoon appointment. Upon dental exam, she had mucosal irritation and erosion of the mucosa at the tongue base and gingiva and a chamomile mouthwash was recommended. She had previously been treated with elmex® fluid (1 % fluoride dental solution) in the past without problems, but it had not been elmex® gelée. The dentist was suspicious of an allergy to the "arome content" of the product. Use of the product was discontinued on 09Dec2010. The patient has recovered from all events.

Case ID	Source	Product
[redacted]	Spontaneous	elmex® gelée

Initial information was received in [redacted] on 16Jan2012 from a male consumer. This consumer used Elmex (Unspecified product in the initial report) and experienced allergic shock. The event occurred on 12Jan2012 and he received emergency treatment. He continued to use the gel, and he increased it. He consulted a healthcare professional but no information was provided. Follow-up information was received on 02Mar2012. The consumer, had history of multiple allergies and the product was identified as Elmex Fluoride Therapy Gel. He began using the gel in mid Dec2011, applying about 10 pieces (small amounts on dentist recommendation) three times per week. The consumer had recovered. He treated himself with dimetindene plus betamethasone.

On 21Mar2012, the dentist confirmed that the event was possibly related to the Elmex product and recommended that the patient should not use Elmex Gel anymore.

Additional information was received on 23Mar2012 from a general doctor. The patient, with a history of multiple allergies and asthma, saw the doctor on 13Jan2012 for breathing problems. The patient attributed the problems to the gel which he used the day before the visit. No emergency treatment was given (contradictory with the patient report) and the clinical checkup was uneventful. The event of allergic shock could not be confirmed or denied by the doctor.

Case ID	Source	Product
[redacted]	Spontaneous	elmex® gelée

Initial information was received, in [redacted] on 12Jul2010, from the [redacted] egarding a female consumer. This 25 year old consumer, with a history of multiple allergies, used elmex® gelée and experienced massive inflammation that included redness, burning, itching and swelling of the oral mucosa. In addition, she had partial exfoliation of the oral mucosa, pain, and a feeling of numbness. She first used the product in May2010. The product was used once for five minutes and was applied with a toothbrush. She used the recommended dose as per the package insert. The consumer did not rinse her mouth after using the product. The symptoms occurred a few hours after the first use and lasted four to five days. She did not consult a healthcare professional regarding these events. The consumer discontinued use of the product. At the time of the report, the consumer had improved completely. Per the [redacted] this case was considered serious and causality was very probable.

6.4.2 Serious Listed Reports

One medically confirmed serious listed report of adverse reactions has been received during the period of this PSUR from a regulatory authority:

Case ID	Source	Product
[redacted]	Spontaneous	elmex® gelée

Initial information was reported, i[redacted]on 12Oct2009, from a physician to [redacted] concerning a 75 year old male consumer who used Elmex Fluoride Therapy Gel and experienced swollen and red gums, mouth edema and stomatitis. The events began on 08Sep2009 and use of the toothpaste was discontinued. At the time of the report, the consumer's outcome was recovering. No further information was obtained.

6.4.3 Non-Serious Unlisted Reports

In the reporting period of this PSUR, 30 non-serious case reports with at least one unlisted symptom were reported by health care professionals or competent authorities. Most of the reactions involve minor reactions in the oral cavity, quite usual for the type of products.

Case reports involving less usual reactions have been selected for a more detailed description although, for most of them, the causal relationship with the use of the products is not clearly established.

There were 6 cases reporting tooth discolouration. One of these is described below:

Case ID	Source	Product
[redacted]	Spontaneous	elmex® gelée

B

Initial information was received on 07Mar2012 from a dentist. A female patient, a child of unknown age, used Elmex Fluoride Therapy Gel and experienced a yellow discoloration of her teeth. She began using the dental gel in February 2012, approximately three weeks prior to the report, brushing with a normal amount once a week. Subsequently, at approximately the same time, she developed the event. She reduced the amount of toothpaste she was using (date and amount not specified). She used the toothpaste for three weeks, but the final action taken with the toothpaste was unknown. She was scheduled to visit the dentist on 07Mar2012. The dentist did not know if the event was causally related to the gel or not. The outcome was unknown.

There were 2 cases with a complaint of tooth pain in two children. One of the cases is described below:

Case ID	Source	Product
[redacted]	Spontaneous	elmex® gelée

Initial information was received in [redacted] on 01Sep2011 from a school dental service nurse. A female child was administered Elmex Fluoride Therapy Gel and developed heavy tooth pain. Therapy had been initiated two years prior to the report (2009) as part of a school dental service. The amount used was not specified, but it was in accordance with the prescription and was applied once every two months. Every time the product was applied, the child experienced the event. Each time, the pain wore off within an hour. The dental gel had been used for two years, and therapy was ongoing. The dose had not been reduced and no healthcare professional had been consulted. The nurse assessed causality as unknown. On 21Oct2011, the nurse was contacted for additional information, but none was available. The outcome of the event was unknown. B

Most of other cases were related to moderate allergic reactions or local irritation in the oral cavity as documented in the following examples:

Case ID	Source	Product
[redacted]	Spontaneous	elmex® gelée

B

Initial information was received on 09Jan2012 from a healthcare professional regarding a female patient. The patient used Elmex Fluoride Therapy Gel and experienced mouth and lip swelling as well as itchy lips. She began using the gel four days prior to this report, applying a pea sized amount. She used the gel only once and the events occurred that same day. The patient discontinued use of the gel that same day and it is unknown if she

planned on restarting use. The reporter did not know if the events were related to use of the gel. It was unknown if the patient recovered from the events.

Case ID	Source	Product
[redacted]	Spontaneous	elmex® gelée

Initial information was received on 17Jan2012 from a healthcare professional. A male patient used Elmex Fluoride Therapy Gel and developed dryness of the skin and lips. He also experienced dryness and bleeding of the oral mucosa. He began using the dental gel and experienced the events on unspecified dates. The patient consulted a healthcare professional. The reporting healthcare professional considered the events related to the dental gel. The outcome of the event was unknown.

Case ID	Source	Product
[redacted]	Spontaneous	elmex® gelée

Initial information was received in [redacted] on 23Jan2012 from a dentist. A male patient used Elmex Fluoride Therapy Gel and developed gum redness, a swollen tongue, and throat burning. He began using the dental gel on an unspecified date, applying the regular amount for use with a bite (applicator) for four minutes once a day. Subsequently, on an unknown date, he experienced the events. He stopped using the dental gel the same day that the event occurred, using the product once and the events resolved (dates unknown). No treatment was administered. The dentist considered the events related to use of the dental gel. The consumer had recovered from the events.

Case ID	Source	Product
[redacted]	Spontaneous	elmex® gelée

Initial information was received in [redacted] on 06Mar2012 from a dental hygienist. The patient received an application of Elmex Fluoride Therapy Gel and showed signs of throat swelling and respiratory problems. On 02Mar2012, the patient received a regular amount of the gel for four minutes and it was applied once only. The events occurred on 02Mar2012 and lasted for three minutes. The patient had recovered. The dental hygienist confirmed that the events were possibly related to the product.

Case ID	Source	Product
[redacted]	Spontaneous	elmex® gelée

Initial information was received in [redacted] on 26Nov2009 from a pharmacist. A female patient used Elmex Fluoride Therapy Gel (Lot # 92091G), applying the product to her thumb and on the forefinger but not her mouth or teeth. She experienced redness, burning and pain. In addition, she developed blisters to her thumb and forefinger. Her blisters were two centimeters in size and they opened and bled. She discontinued use of the product on an unspecified date. The patient was recovering, as the healing process was nearly complete.

Case ID	Source	Product
[redacted]	Spontaneous	elmex® gelée

Initial information received in [redacted] on 18May2011 from a pharmacist. A female patient used Elmex Fluoride Therapy Gel(Lot #10382G) and experienced facial swelling. She used the product once and experienced the event immediately after the application. She spoke with a healthcare professional concerning the event. The consumer had recovered, as her condition was stable.

6.4.4 Non-Serious Listed Reports

In the reporting period of this PSUR, there were 24 spontaneous and medically confirmed non-serious listed case reports. Most of them were mild reactions in the oral cavity as could be expected for this type of product and there was one case of asymptomatic accidental exposure (see Annex 1).

6. 5 All Death Cases

No fatal cases related to the use of elmex® gelée were reported.

7. STUDIES

7.1 Newly analyzed Company-Sponsored Studies

There has been no sponsored safety studies carried out on elmex® gelée during the period under review.

7.2 Targeted New Safety Studies

There are no new targeted safety studies planned on elmex® gelée.

7.3 Published Safety Studies

A search has been carried out for any independent published studies cited in medical and scientific journals where the product or its ingredient may have been used in safety studies/clinical trials.

There were no publications related to Elmex® gelee or to amine fluoride and bringing new information about the safety of the product.

References and summaries for these published studies are provided in Appendix 9.

7.4 Other Studies

There have been no specific studies carried out on pregnant patients and no relevant safety information was reported related to pregnancy exposure during the period of this report.

8. OTHER INFORMATION

Since the data-lock point, no relevant new information that might affect the interpretation or evaluation of existing reports has come to our knowledge.

8.1. Lack of efficacy

No cases of lack of efficacy were referred to us during the period of this report.

8.2. Late breaking information.

Since the data-lock point, no relevant new information that might affect the interpretation or evaluation of existing reports has come to our knowledge.

8.3 Risk Management Plan

No Risk management Plan is in place for this product.

8.4 Risk-Benefit Analysis Report

No specific risk-benefit analysis has been conducted on this product. However, no changes in the benefit of the product, no relevant changes in the type or frequency of adverse events and no new information from literature or studies which could affect the safety risk of the product were identified. Therefore, it may be considered that the risk-benefit balance of the product is not modified.

9. OVERALL SAFETY EVALUATION

The information gathered during the period of review is principally consistent with the established safety profile of elmex® gelée.

9.1 Cumulative Perspective: Serious Unlisted Reactions

There were 4 spontaneous unlisted serious cases reported during the period of this PSUR. There were no other spontaneous serious unlisted cases reported prior to this PSUR.

From these 4 reports, a total of 29 MedDRA coded serious unlisted adverse reactions were collected till 31 July 2012. None of the unlisted terms appeared more than twice. As a consequence, the occurrence rate of each of the serious unlisted adverse reports remains very low. (see Appendix 6)

9.2 Cumulative Perspective: Serious Listed Reactions

There was one medically confirmed serious listed cases reported by a Regulatory Agency during the period of this PSUR. There was no other case prior to the period of this PSUR. (see Appendix 7)

9.3 Cumulative perspective: Non-Serious Unlisted Reactions

There were 30 non-serious unlisted case reports during the reporting period and 48 non-serious unlisted preferred MedDRA coded terms (Appendix 8). Tooth discolouration, being the most frequently reported event, appeared 6 times within this reporting period. For this term, this represents an occurrence rate of 1 report per approximately [redacted] packs sold. A

9.4 Increased Reporting Frequency of Listed Reactions

A total of 77 non-serious listed reactions were reported during the period of this PSUR. There were 24 spontaneous non-serious listed case reports which generated 55 non-serious listed reactions described by their MedDRA coded Preferred Terms in Annex 1. 22 non-serious listed reactions were also reported from the non-serious unlisted cases (Appendix 5).

Oral pain occurred 5 times. Oral mucosal exfoliation was reported 4 times. Gingival pain, Lip swelling, Oral discomfort and Oral mucosal blistering appeared 3 times; Stomatitis, Swollen tongue, Aphthous stomatitis, Cheilitis occurred twice while other reactions appeared only once. For all of them, the occurrence rate remains low for the listed reactions As compared to the overall exposure to the product.

9.5 Changes in Characteristics of Listed Reactions

None of the listed reactions observed during the reporting period may be considered as a signal requiring a change in the list of potential undesirable effects as they were either isolated cases and/or insufficiently related with certainty to the use of elmex® gel.

9.6 Interactions

There were no reports of drug interactions with elmex® gelée.

9.7 Experience with overdose

No cases of experience with overdose were referred to us during the period of this report.

9.8 Abuse and misuse

There was one report of misuse

Case ID	Source	Product
[redacted]	Spontaneous	elmex® gelée

B

Initial information was received in [redacted] on 27Mar2012 from a pharmacist. The consumer's daughter reported that her father used Elmex Fluoride Therapy Gel and experienced cauterization in the mouth. He began using the gel on an unspecified date (dose/frequency unknown). On 23Mar2012, he left the gel in his mouth overnight. The next day on 24Mar2012, he experienced the event. The outcome was unknown.

9.9 Experience with pregnancy and lactation

During this reporting period, there is one case of exposure during pregnancy; however there was no adverse event and the case was also not medically confirmed.

9.10 Experience in special patient groups

The distribution of medically confirmed ADRs was as follows:

Category	Related age	Number of ADRs
Newborn infants	0-27 days	0
Infants	28 days – 23 months	1
Children	2 – 11 years	6
Adolescent	12 – 16 years	4
Adults	17 – 64 years	10
Seniors	65 years and more	5
Not indicated		33

Gender	Number
Male	20
Female	28
Not indicated	11

In approximately 55% of the reports, the age was not indicated and in 19% of the reports, gender was not specified. The incident rate represented for each age category shows a majority of cases reported from adult patients as expected and does not identify any age-related specific type of reaction.

In terms of the distribution of the reactions between patients gender, there were no meaningful differences between women and men.

Overall, reactions on these patient groups did not provide any specific signals for the respective populations.

9.11 Effects of long-term treatments

No cases were reported for effects of long-term treatments during the period of this report.

9.12 Cases from non-health care professionals

Fifty one (51) case reports were collected directly from consumers/patients during the period of this report and a line listing with summary tabulation by SOC is presented as Annex 2. A total of 139 adverse reactions were reported from which a majority (62) were related to gastrointestinal disorders as could be expected for toothpaste. As usually expected for consumer/patient reports, most of them do not provide sufficient evidence of a direct causal relatedness with the use of the product.

One of the 51 cases was considered serious by the MAH and is described below:

Case ID	Source	Product
[redacted]	Spontaneous	elmex® gelée

B

Initial information was received in [redacted] on 11Aug2011 from a consumer's mother. This 26 month old male stood on a stool, located and swallowed a large amount of elmex® gelée, and had an allergic reaction. As a result, he vomited, his lips turned blue and he turned white. In addition, his forehead swelled up a little and became red, and he became very tired. He ingested the dental gel on 08Aug2011 but the exact time was unknown. The reporter noted that the cap was not sealed and therefore, her son was able to open the tube as a result. She closed the tube after the ingestion occurred. The reporter stated that her son did not admit to ingesting the dental gel, but she was certain he did because his vomit smelled like elmex® gelée. Immediately after the events occurred and prior to going to the hospital, the consumer breastfed her son and gave him calcium. He was taken to the hospital the evening of 08Aug2011, where he was placed on machines. Subsequently, he recovered from the events but was kept overnight for surveillance. On 09Aug2011, the consumer breastfed her son again and he was discharged at 3:00 pm. The reporter was not certain of the approximate amount of dental gel that was in the tube prior to ingestion. The consumer did not provide contact information for the healthcare professionals.

Overall, the consumer reports did not bring any new information to the safety assessment of elmex® gel when used according to the SPC recommendation.

9.13 Prescription errors/medication errors

There were no cases of prescription error during the period of the report.

10. CONCLUSIONS

The experience gained during the period covered by this report confirms the established good safety profile of elmex® gelée and that the risk-benefit profile has not changed.

Overall, the Summary of Product Characteristics contains sufficient information to inform physicians, dentists, pharmacists and patients about the occurrence of adverse drug reactions and to warrant the safe use of elmex® gelée which still has an excellent risk-benefit ratio when used under the conditions stipulated in the Summary of Product Characteristics.

APPENDIX 1 - Worldwide Marketing Authorisation Status

Country	Register-No.	Date of Registration	Date of Last Renewal	Regulatory Status: Drug	MAH	Trade Name	Indications
Switzerland	34916 (02/039)<br>34916 (02/047)	17.06.1969<br>14.09.1995	Application 7.3.2008	25 g tube: OTC<br>215 g tube: OTC	GABA International AG	elmex® gelée	Prevention of caries.<br>To support the treatment of initial caries.<br>Treatment of sensitive dental necks.
Germany	6169101.00.00<br>former E 1082	18.12.1970	11/2004<br>ongoing	25 g tube: OTC<br>38 g, 215g tube: Rx	GABA GmbH	elmex® gelée	For prevention of caries, particularly in children, adolescents and patients with dental braces, other orthodontic appliances and partial dentures.<br>To support the treatment of early caries; for treatment of hypersensitive dental necks
The Netherlands	RVG 06269<br>RVG 09027	18.07.1973<br>01.04.1982	27.10.2006 approved<br>05.10.1999 Registration withdrawn	38 g tube: Rx<br>25g tube: OTC<br>Formula 447/1761	GABA B.V.	elmex® medical cariesprotectie gel 12,5 mg/g, tandgel	Prevention of dental caries in case of high caries risk<br>Decalcification of the enamel under anchors of partial removable protheses or under orthodontal devices<br>Refluoridation of tooth enamel of polished surfaces
Finland	6737	20.03.1974	12.2.2008	25 g Tube: Rx<br>215 g tube : Rx	GABA GmbH	elmex® dentaaligeeli	See Germany
Croatia	UP/I-530-09/05-02/99	10.12.1980	27.01.2005 Registration withdrawn in 2009 Submission for new registration 17.12.2010 (pending) current import licence	25 g tube: OTC	Belupo Ltd.	Aminfluorid zele	See Germany
Slovenia	4-120-025	1980 Yugoslavia	24.10.2005	25 g tube: Rx Registration withdrawn in 2007	Belupo Ltd.	elmex® gelée	
Spain	263-Dent	09.02.1981	06.02.2009approved	25 g tube: Dentifrice	GABA GmbH	elmex® gel	See Germany
Czech Republic	95/006/82-S C	28.01.1982	09.06.2010	25 g tube: OTC<br>215 g tube: Rx	GABA GmbH	elmex® gelée	Caries prevention and fluoridation of tooth enamel<br>Treatment of early stages of caries (remineralisation)<br>Prophylaxis and care of sensitive teeth
Slovak Republic	87/0006/82-S	28.01.1982	31.01.2003	25 g, 215 g tube: OTC	GABA GmbH	elmex® gelée	See Germany
Israel	064 99 21609 00	30.06.1982	8.8.2004	25 g tube: Rx	Salamon, Levin & Elstein Ltd	elmex® gelée	Routine prophylaxis against caries, individual and collective.<br>Cases with a high tendency for caries.<br>Treatment of sensitive dental necks

Belgium	1362 LC 1 F7	24.09.1982	29.09.2004	25 g tube: OTC<br>38 g tube: OTC<br>215 g: Rx (hospital)	GABA B.V.	elmex® medical gel	elmex Medical Gel is indicated in the prevention of dental caries.<br>elmex Medical Gel can be used by subjects wearing orthodontic devices, on the juxtadental surfaces of partial prostheses and removable wire splints and for the treatment of sensitivity of the dental neck.
Luxembourg	0311/00056736	07.07.1983	24.10.2002	25 g<br>38 g: Rx<br>215 g: Rx	GABA GmbH	elmex® gelée	See Germany
South Africa	X596	1986	No specific date	25 g tube: OTC	Dental Warehouse (distributor)	elmex® gelée	The prevention of dental caries<br>The treatment of incipient caries lesions (remineralisation)<br>The treatment of hypersensitive teeth
Austria	1-18093	03.06.1986	01.06.2006	25 g tube: OTC	Gebro Pharma GmbH	elmex® Zahngel	Treatment of initial caries lesions (after the remaining teeth have erupted) in conjunction with dietary advice and oral hygiene<br>Surface mineralisation of sensitive dental necks<br>Decalcification of enamel underneath removable bridges,<br>Refluoridation of worn sections of enamel<br>Selective grinding and dental enamel lesions
Italy	0264870 13<br>0264870 25	19.06.1987	26.09.2009 ongoing	25 g tube: Categoria C (without prescription)<br>215 g tube: Rx	GABA Vebas srl.	elmex® gel	1. Caries prophylaxis; hypersensitivity of dental necks<br>2. Decalcification of dental enamel caused by removable splints, partial prostheses and orthodontic appliances<br>3. Refluoridation of dental enamel

Poland	R/0676	21.06.1990<br>22.12.2000<br>22.07.2005	12.02.2008 approved	25 g tube: OTC<br>215 g tube: OTC<br>38g tube: OTC	GABA GmbH	elmex® zél	prophylaxis:<br>o intensive dental caries prevention, especially suitable for children, adolescents and groups who are particularly at risk of developing caries – patients with orthodontic braces, bridges or partial dentures treatment:<br>o the remineralisation of initial caries lesions<br>o the treatment of tooth neck hypersensitivity (see Germany)
Hungary	OGY-T-1646/01<br>OGY-T-1646/02	03.07.1991	22.11.2007 approved	25 g tube: OTC<br>215 g tube: OTC	Teva Hungary Ltd.	Elmex®® gelée	Caries prevention and fluoridation of tooth enamel<br>Treatment of early stages of caries (remineralisation)<br>Prophylaxis and care of sensitive teethPrevention (See Czech Rep.)
Portugal	2523397	31.10.1996	31.10.2006 on going	25 g tube: OTC	GABA GmbH	elmex® gel	1. Caries prophylaxis 2. Hypersensitivity

APPENDIX 2
Company Core Data Sheet (CCDS)

1. NAME OF THE MEDICINAL PRODUCT

elmex® gelée, dental gel

2. QUALITATIVE AND QUANTITATIVE COMPOSITION

Active ingredients, qualitative and quantitative
1g elmex® gelée contains:

Amine fluoride:

Dectaflur	2.87 mg
Olaflur	30.32 mg
Sodium fluoride	22.10 mg

This corresponds to a fluoride content of 1.25%.
For excipients, see 6.1.

3. PHARMACEUTICAL FORM

Dental gel

4. CLINICAL PARTICULARS

4.1 Therapeutic indications

Prophylaxis
For caries prophylaxis as well as patients with dental braces, other orthodontic appliances and partial prostheses.

Therapy
Adjunctive treatment of initial caries; and treatment of hypersensitive dental necks.

4.2 Posology and method of administration

To be applied on the teeth.
The following doses are recommended. The dosage may be increased in times when there is a greater risk of caries and for the treatment of hypersensitive dental necks. This applies particularly to patients with orthodontic appliances.

Use at home
elmex® gelée should not be used before swallowing reflex is fully developed (e.g. children under sic years of age or disabeled people?
Use once a week.Apply about 1-2 cm elmex® gelée (approx..0.5 g dental gel corresponding to 6.25 mg fluoride) to a toothbrush and brush the teeth. Rinse after 2 to 3 minutes. Best used in the evenings just before going to sleep. For targeted treatment of hypersensitive dental necks, elmex® gelée is applied to the affected surface with as oft brush and gently rubbed in.

The total time of application (brushing and residence time) must not exceed 5 minutes.

Professional use

elmex® gelée is used with an appropriate gel carrier (miniplast splints or spoon applicators) or applied directly to the masticatory surfaces and interdental spaces with the blunt cannula of a filled disposable syringe.
Adequate contact time of the dental gel with the teeth (at least 2 to 4 minutes) must be maintained. Do not, however, exceed 5 minutes. Rinse out the mouth after use.

Use elmex® gelée about twice a year as part of dental treatment or in individual caries prophylactic activities, or more often in high-risk patients:
- in miniplast splints approx. 3 g elmex® gelée, corresponding to approx. 37.5 mg fluoride;
- in spoon applicators up to 8 g elmex® gelée, corresponding to up to 100 mg fluoride;
- with the blunt cannula of a filled disposable syringe, apply directly to the masticatory surfaces and inter dental spaces (0.5 to 1 g elmex® gelée, corresponding to 6.25 to 12.5 mg fluoride).

Spoon application is indicated from 8 years of age.

Use in the group prophylaxis (applicable for all countries (D, CH, PL) where school has prophylaxis program)

Elmex® gelée is a suitable fluoridation form for in class intensive fluoridation in the context of school dental prophylaxis programs. Frequency of application should be determined by the appropriate national association , a suggested range of 2-4 times a month was shown to be most effective. The method of application should be as use at home or if done by a professional by minplast splints or spoon applicators.
Rinse out the mouth after use. The total time of application (brushing and residence time) must not exceed 5 minutes.

4.3 Contraindications

elmex® *gelée* must not be used in cases of:

- hypersensitivity to any one of the components;

- pathological desquamative changes of the oral mucosa (erosion of the epithelium).
- In people whose swallowing reflex is impaired or not yet fully developed
- In case of bone and/or enamel fluorosis

4.4 Special warnings and special precautions for use

elmex gel is not suitable for use in people from whom control of the swallowing reflex cannot be guaranteed (e.g. children under six years of age, disabled persons)

Due to the risk of overdose and subsequent intoxication the application of elmex® gelée in the Miniplast tray or with a suitably moulded wax tray is not recommended in children under eight years of age.

After spoon application at short intervals exfoliation (desquamation), superficial defects (erosions) und ulcers of the oral mucosa have been observed in very rare cases.

Because of peppermint oil and spearmint oil contained in the gel, patients with bronchial astma or other airway disorderd should speak to theire doctor/dentist before using elmex gel.

4.5 Interaction with other medicinal products and other forms of interaction

Systemic supplies of fluoride (e.g. with fluoride tablets) should be stopped for a few days after the application of elmex® *gelée.*

4.6 Pregnancy and lactation

There are no or limited amount of data (less than 300 pregnancy outcomes) from the use of Fluorides in pregnant women. Animal studies do not indicate direct or indirect harmful effects with respect to reproductive toxicity (see section 5.3). As a precautionary measure, it is preferable to avoid the use of elmex gele during pregnancy.] Fluorides have been identified in breastfed newborns/infants of treated women. There is insufficient information on the effects of Fluorides in newborns/infants.

4.7 Effects on ability to drive and use machines

Elmex gel has no influence on the ability to drive and use machines.

4.8 Undesirable effects

Gastrointestinal disorders (very rare < 1/10 000)

- Oral mucosal exfoliation
- Oral irritation (stomatitis/redness, oral discomfort/burning, numbness, swelling/oedema, inflammation, oral pruritus, altered taste, mouth dryness, gingivitis)
- Superficial oral mucosal erosion/mouth ulceration (ulcers, sores, blisters)

- Nausea or vomiting

Immune system disorders (very rare < 1/10 000)

- Hypersensitivity (allergic reactions)

4.9 Overdose

a) Symptoms of overdose

acute:

Local irritation of the mucosa is possible in cases of acute overdosage.
Depending on the dose and method of application, in extreme cases (e.g. with spoon application) up to 100 mg fluoride, corresponding to 8 g elmex® *gelée*, may be introduced into the oral cavity. Swallowing this amount may give rise to nausea, vomiting and diarrhoea. In most cases, these symptoms occur within the first hour after ingestion and resolve within three to six hours.

chronic:

Regularly exceeding a total daily fluoride dose of 2 mg during the development of the teeth up to approximately 8 years of age may lead to disturbances in the mineralisation of the dental enamel. It appears as flecks on the dental enamel. This condition, known as dental fluorosis, no longer occurs after this age, even at high daily doses.

b) Management of overdosage

acute:

With mild symptoms of intoxication (less than 150 mg fluoride, corresponding to less than 12 g elmex® *gelée*) calcium-containing drinks (milk, soluble calcium tablets) should be given to bind the fluoride.

With severe symptoms of intoxication (more than 150 mg fluoride, corresponding to more than 12 g elmex® *gelée*) the additional administration of activated charcoal is recommended. If necessary, calcium may be given intravenously, forced diuresis with alkalinisation of the urine may also be initiated. Heart rate, coagulation, electrolyte and acid-base balance should be monitored carefully.

5. PHARMACOLOGICAL PROPERTIES

5.1 Pharmacodynamic properties

Agent for caries prophylaxy
ATC code: A01A A51

The caries protection and therapeutic effects of fluorides can be attributed to three factors:

1. The increase in acid resistance of the dental enamel.
2. Inhibition of sugar breakdown by acid-producing micro-organisms in dental plaque.
3. Promotion of the remineralisation of initial carious lesions.

In amine fluoride containing compounds, the cation strengthens the caries protection and therapeutic effects. Polarisation between the hydrophobic long-chain alkyl residues and the hydrophilic amine groups confers surfactant properties to the cation. In particular, these are:

1. Longer retention time of the fluoride in the oral cavity.

2. Wetting of the clinical crown of the tooth.

3. Good ability to react with the dental enamel, which allows chemical changes to take place after only seconds:
   incorporation of fluoride into the enamel (stable fluoride reservoir) and formation of a labile fluoride reservoir (calcium fluoride coating layer).

4. Marked affinity to dental plaque, which leads to slightly raised fluoride concentrations and in particular to a longer retention time of fluoride in the plaque.

5. Antimicrobial properties.

6. Inhibition of bacterial sugar breakdown to acids, lasting several hours.

7. Improved adherence of the fluoride-rich coating layer on the enamel surfaces, which can be seen in an increased acid resistance.

Treatment of initial enamel lesions through remineralisation of already decalcified areas of enamel (incipient caries) is improved by the presence of fluoride ions, in that more phosphate and calcium from the saliva are again deposited in the partially demineralised enamel. This reaction is very effectively promoted through the amine fluoride contained in elmex® *gelée*, which remains on the tooth surfaces for a long time.

For caries prophylaxis and adjunctive treatment of initial caries, the formation of a very fluoride-rich, adherent yet adequately labile coating layer is of particular importance, so that the fluoride ions may be released over weeks or even months in concentrations such as those found after the consumption of fluoridated drinking water.

Elmex® *gelée* also forms a calcium-fluoride coating layer on exposed dentine, which covers or obliterates the openings of the dentinal tubules. This protective film prevents the transmission of external stimuli from the oral cavity, thus desensitising the hypersensitive dental necks. Pain relief is not permanent and the desensitisation must be repeated as required.

5.2 Pharmacokinetic properties

Fluoride concentration profiles in the serum following topical application of fluoride-containing dental gels differ from the concentrations seen after ingestion, i.e. oral doses not coming into contact with the tissues of the oral cavity.

Depending on the mode of application (toothbrush, use of miniplast splints or spoon applicators), the retention capacity of the dentition (affected by positioning, dentures, salivary flow), material-specific characteristics (adhesiveness, surface affinity) as well as further individual factors (e.g. consumption of food and drink) the fluoride retained in the oral cavity after topical application is desorbed from its site, swallowed and absorbed in varying amounts at different times. It is therefore not possible to obtain data on the time and intensity of peak concentrations.

The pharmacokinetic properties of orally administered fluorides are well researched. At low pH values, fluoride is converted into non-dissociated HF molecules which are rapidly absorbed. Fluoride is quickly and completely absorbed from the small intestine. The peak plasma concentration is achieved within 30 minutes. Plasma half-life is about three hours (1.5 – 5 hours). Fluoride is mainly eliminated via the kidneys. Very small quantities (insoluble calcium salts) are excreted in the faeces. The greater the rate of diuresis and the greater the alkalinity of the urine, the faster the rate of renal excretion of fluorides. Fluorides are released in the saliva and re-absorbed in the gastrointestinal tract. Fluorides are also excreted in breast milk.

Fluoride is a naturally-occurring component of the body and is found in bones and the hard substances of the teeth. Prolonged daily ingestion of excessive fluoride may result in varying degrees of fluorosis. By the age of 8, there is no longer a risk for dental fluorosis. Doses of fluoride associated with dental fluorosis and risk of bone fracture would be well above the expected exposure level from elmey gele.

5.3 Preclinical safety data

Non-clinical data reveal no special hazard for humans based on conventional studies of safety pharmacology, repeated dose toxicity, genotoxicity, carcinogenic potential and toxicity to reproduction and development.

6. PHARMACEUTICAL PARTICULARS

6.1 List of excipients

Purified water, propylene glycol, hydroxyethylcellulose, saccharin, apple flavour, peppermint oil, spearmint oil, menthone flavour, banana flavour

6.2 Incompatibilities

The ingestion of calcium, magnesium (e.g. milk) and aluminium (in medicines for the treatment of stomach problems; antacids) salts immediately following treatment with elmex® gelée may reduce the effects of the fluorides.

Incompatibilities also exist with anionic tensides and other large anionic molecules.

6.3 Shelf life

3 years

After opening the container, the medicinal product is to be used until the end of the expiry date.

6.4 Special precautions for storage

Do not store over 25°C

6.5 Nature and contents of container, package sizes

25g tube: Polyethylene laminate stand tube with a child-proof closure.
38g and 215g tube: Polyethylene laminate stand tube.

6.6 Instructions for use and handling

No special requirements.

7. MARKETING AUTHORISATION HOLDER

Corporate Headquarter: GABA International Ltd.
Emil Frey Strasse 100, CH-4142 Münchenstein, Switzerland

8. MARKETING AUTHORISATION NUMBER(S)

See List of World Wide Marketing Authorisation, in section 2 of the PSUR.

9. DATE OF FIRST AUTHORISATION/RENEWAL OF THE AUTHORISATION

See List of World Wide Marketing Authorisation, in section 2 of the PSUR.

10. DATE OF REVISION OF THE TEXT

Version 07
Therwil,21.08.2012

Document History

Version	Changes
Version 07	- Posology and Method of administration: rearrangement<br>- Contraindication: addition of two additional CI<br>- Special warnings and precautions are rearranged and reworded and one warring is added "Because of peppermint oil and spearmint oil contained in the gel, patients with bronchial asthma or other airway disordered should speak to their doctor/dentist before using elmex gel."<br>- Interactions: rearrangements<br>- Pregnancy and Lactation: use of QRD template<br>- Effects on ability to drive: clarification<br>- Undesirable effects: use of MEDRA nomenclature and frequency, addition of UEs<br>- Preclinical Safety: additional data, use of QRD template<br>- Incompatibilities: rearrangements

APPENDIX 3
Company Core Safety Information (CCSI)

NAME OF THE MEDICINAL PRODUCT

CH, GE, Curacau, SLO, CZ, SK, Isr, Lux, Sth Afr, HU, Sth Ko,	elmex® gelée
NL	elmex® medical cariesprotectie gel 12,5 mg/g, tandgel
FN	elmex® dentaaligeeli
Cro	Aminfluorid zele
BE	Gel médical elmex®
Austria	elmex® Zahngel
IT	elmex® gel dentale.
PL	elmex® zél
PT, SP	elmex® gel

Posology and Method of Administration (Ref 4.2 of CCDS)

To be applied on the teeth.
The following doses are recommended. The dosage may be increased in times when there is a greater risk of caries and for the treatment of hypersensitive dental necks. This applies particularly to patients with orthodontic appliances.

Use at home
elmex® gel should not be used before swallowing reflex is fully developed (e.g. children under six years of age or disabled people)
Use once a week. Apply about 1-2 cm elmex® gel (approx. 0.5 g dental gel corresponding to 6.25 mg fluoride) to a toothbrush and brush the teeth. Rinse after 2 to 3 minutes. Best used in the evenings just before going to sleep.
For targeted treatment of hypersensitive dental necks, elmex® gel is applied to the affected surfaces with a soft brush and gently rubbed in.

The total time of application (brushing and residence time) must not exceed 5 minutes.

Professional use
elmex® gel is used with an appropriate gel carrier (miniplast splints or spoon applicators) or applied directly to the masticatory surfaces and interdental spaces with the blunt cannula of a filled disposable syringe.
Adequate contact time of the dental gel with the teeth (at least 2 to 4 minutes) must be maintained. Do not, however, exceed 5 minutes. Rinse out the mouth after use.
Use elmex® gel about twice a year as part of dental treatment or individual caries prophylactic activities, or more often in high-risk patients:
- In miniplast splints approx. 3 g elmex® gel, corresponding to approx. 37.5 mg fluoride;
- In spoon applicators up to 8 g elmex® gel, corresponding to up to 100 mg fluoride;
- With the blunt cannula of a filled disposable syringe, apply directly to the masticatory

surfaces and interdental spaces (0.5 to 1 g elmex® gel, corresponding to 6.25 to 12.5 mg fluoride).
Spoon application is indicated from 8 years of age.

Use in the group prophylaxis (applicable for all countries where school has a prophylaxis program)
elmex® gel is a suitable fluoridation form for in class intensive fluoridation in the context of school dental prophylaxis programs. Frequency of application should be determined by the appropriate national dental association, a suggested range of 2-4 times a month was shown to be most effective. The method of application should be as the use at home or if done by a professional by miniplast splints or spoon applicators.

Rinse out the mouth after use. The total time of application (brushing and residence time) must not exceed 5 minutes.

Contraindications (Ref 4.3 of CCDS)

elmex® gel must not be used in cases of:

- Hypersensitivity to any one of the components;
- Pathological desquamative changes of the oral mucosa
- In people whose swallowing reflex is impaired or not yet fully developed
- In case of bone and/or enamel fluorosis

Special Warnings and Precautions for Use (Ref 4.4 of CCDS)

elmex® gel is not suitable for use in people for whom control of the swallowing reflex cannot be guaranteed (e.g children under six years of age, disabled persons)

Due to the risk of overdose and subsequent intoxication the application of elmex® gel in the Miniplast tray or with a suitably moulded wax tray is not recommended in children under eight years of age.

After spoon application at short intervals exfoliation (desquamation), superficial defects (erosions) and ulcers of the oral mucosa have been observed in very rare cases

Because of the peppermint oil and spearmint oil contained in the gel, patients with bronchial
asthma or other airway disorders should speak to their doctor/dentist before using elmex gel.

Interaction with other Medicinal Products and other forms of Interaction (Ref 4.5 of CCDS)

Systemic supplies of fluoride (e.g. with fluoride tablets) should be stopped for a few days after the application of elmex® gel.

Fertility, Pregnancy and Lactation (Ref 4.6 of CCDS)

There are no or limited amount of data from the use of fluorides in pregnant women. Fluorides are excreted in human milk and a decision must be made whether to discontinue brest-feeding or to discontinue from intensive fluoridation therapy taking into account the benefit of breast feeding for the child and the benefit of therapy for the woman.

Effects on Ability to Drive and Use Machines (Ref 4.7 of CCDS)

elmex gel has no influence on the ability to drive and use machines.

Undesirable Effects (Ref 4.8 of CCDS)

Gastrointestinal disorders
- Oral mucosal exfoliation
- Oral irritation (stomatitis/redness, oral discomfort/burning, numbness, swelling/oedema, inflammation, oral pruritus, altered taste, mouth dryness, gingivitis)
- Superficial oral mucosal erosion/mouth ulceration (ulcers, sores, blisters)
- Nausea or vomiting

Immune system disorders
- Hypersensitivity (allergic reactions)

Overdose (Ref 4.9 of CCDS)

a) Symptoms of overdose

Acute:
In the case of acute overdose local irritation of the mucosa may occur.
Depending on the dose and method of application, in extreme cases (e.g. with spoon application) up to 100 mg fluoride, corresponding to 8 g elmex® gel, may be introduced into the oral cavity. Swallowing this amount may give rise to nausea, vomiting and diarrhoea. In most cases, these symptoms occur within the first hour after ingestion and resolve within three to six hours.

Chronic:
Regularly exceeding a total daily fluoride dose of 2 mg during the development of the teeth up to approximately 8 years of age may lead to disturbances in the mineralisation of the dental enamel. It appears as flecks on the dental enamel. This condition, known as dental fluorosis, no longer occurs after this age, even at high daily doses.

b) Management of over dosage

Acute:
With mild symptoms of intoxication (less than 150 mg fluoride, corresponding to less than 12 g elmex® gel) calcium-containing drinks (milk, soluble calcium tablets) should be given to bind the fluoride.

With severe symptoms of intoxication (more than 150 mg fluoride, corresponding to more than 12 g elmex® gel) the additional administration of activated charcoal is recommended. If necessary, calcium may be given intravenously, forced diuresis with alkalinisation of the urine may also be initiated. Heart rate, coagulation, electrolyte and acid-base balance should be monitored carefully.

Pharmacodynamic Properties (Ref 5.1 of CCDS)

Agent for caries prophylaxis
ATC code: A01A A51

The caries protection and therapeutic effects of fluorides can be attributed to three factors:

1. The increase in acid resistance of the dental enamel.
2. Inhibition of sugar breakdown by acid-producing micro-organisms in dental plaque.
3. Promotion of the remineralization of initial carious lesions.

In amine fluoride containing compounds, the cation strengthens the caries protection and therapeutic effects. Polarisation between the hydrophobic long-chain alkyl residues and the hydrophilic amine groups confers surfactant properties to the cation. In particular, these are:

1 Longer retention time of the fluoride in the oral cavity.

2 Wetting of the clinical crown of the tooth.

3. Good ability to react with the dental enamel, which allows chemical changes to t place after only seconds: incorporation of fluoride into the enamel (stable fluor reservoir) and formation of a labile fluoride reservoir (calcium fluoride coat layer).

4. Marked affinity to dental plaque, which leads to slightly raised fluor concentrations and in particular to a longer retention time of fluoride in the plaque.

5. Antimicrobial properties.

6. Inhibition of bacterial sugar breakdown to acids, lasting several hours.

7. Improved adherence of the fluoride-rich coating layer on the enamel surfaces, wh can be seen in an increased acid resistance.

Treatment of initial enamel lesions through remineralization of already decalcified areas of enamel (incipient caries) is improved by the presence of fluoride ions, in that more phosphate and calcium from the saliva are again deposited in the partially demineralised enamel. This reaction is very effectively promoted through the amine fluoride contained in elmex® *gel*, which remains on the tooth surfaces for a long time.

For caries prophylaxis and adjunctive treatment of initial caries, the formation of a very fluoride-rich, adherent yet adequately labile coating layer is of particular importance, so that the fluoride ions may be released over weeks or even months in concentrations such as those found after the consumption of fluoridated drinking water.

elmex® *gel* also forms a calcium-fluoride coating layer on exposed dentine, which covers or obliterates the openings of the dentinal tubules. This protective film prevents the transmission of external stimuli from the oral cavity, thus desensitising the hypersensitive dental necks. Pain relief is not permanent and the desensitisation must be repeated as required.

Pharmacokinetic Properties (Ref 5.2 of CCDS)

Fluoride concentration profiles in the serum following topical application of fluoride-containing dental gels differ from the concentrations seen after ingestion, i.e. oral doses not coming into contact with the tissues of the oral cavity.

Depending on the mode of application (toothbrush, use of miniplast splints or spoon applicators), the retention capacity of the dentition (affected by positioning, dentures, salivary flow), material-specific characteristics (adhesiveness, surface affinity) as well as further individual factors (e.g. consumption of food and drink) the fluoride retained in the oral cavity after topical application is desorbed from its site, swallowed and absorbed in varying amounts at different times. It is therefore not possible to obtain data on the time and intensity of peak concentrations.

The pharmacokinetic properties of orally administered fluorides are well researched. At low pH values, fluoride is converted into non-dissociated HF molecules which are rapidly absorbed. Fluoride is quickly and completely absorbed from the small intestine. The peak plasma concentration is achieved within 30 minutes. Plasma half-life is about three hours (1.5 – 5 hours). Fluoride is mainly eliminated via the kidneys. Very small quantities (insoluble calcium salts) are excreted in the faeces. The greater the rate of diuresis and the greater the alkalinity of the urine, the faster the rate of renal excretion of fluorides. Fluorides are released in the saliva and re-absorbed in the gastrointestinal tract. Fluorides are also excreted in breast milk.

Fluoride is a naturally-occurring component of the body and is found in bones and the hard substances of the teeth.

Preclinical Safety Data (Ref 5.3 of CCDS)

Fluoride is not expected to be genotoxic, carcinogenic or teratogenic in humans. The results of genotoxicity testing of sodium fluoride are mixed. However, if genotoxicity was occurring and being manifested in the mammalian systems, it would be expected to be seen as adverse effects on offspring or as tumor formation in chronically exposed animals. The lack of teratogenicity or embryotoxicity and the preponderance of evidence against a carcinogenic effect of fluoride lends greater weight to the absence of effective genotoxicity for fluoride compounds present in elmex® *gel.*

Prolonged daily ingestion of excessive fluoride may result in varying degrees of fluorosis. By age 8, there is no longer a risk for dental fluorosis. Doses of fluoride associated with dental fluorosis and risk of bone fracture would be well above the expected exposure level from elmex® *gel.*

Incompatibilities (Ref 6.2 of the CCDS)

The ingestion of calcium, magnesium (e.g. milk) and aluminium (in medicines for the treatment of stomach problems; antacids) salts immediately following treatment with elmex® gel may reduce the effects of the fluorides.

Incompatibilities also exist with anionic tensides and other large anionic molecules.

DOCUMENT HISTORY

Version	Supersedes Document Number/Active Date	Reason for Revision	List of Changes
-01 to 05	Historical	New and historical updates	01 : initial based on German SmPC<br>02 : change of ATC code<br>03: format change<br>04: add sentence "“The total time of application (brushing and residence time) must not exceed 5 minutes.” Furthermore, additional paragraphs have been introduced for the use of elmex® gel in group prophylaxis<br>05: addition of undesirable effects

-06	Version 05/01-Dec -2011	Harmonization of all CCSI from the company based on most recent safety information and review of Adverse Events including the consumer reports (pro-actively to new PCV regulation 2012).<br>Remove section 4.10 (incorrectly updated) and merge relevant information with section 4.8.<br>Re-formatting of the text to make it more clear<br>Generic name changed from elmex® gelée to elmex® gel	- Harmonization of clinical studies section with other CCSI related to sodium fluoride<br><br>- In section : « Undesirable effects » - revision of Listed AEs based on 5-years HCP and Consumer reports.<br><br>- Minors.<br><br>- Significant re-phrasing<br>- Add Fertility in title of 4.6<br>- In entire document

5. DATE OF REVISION OF THE TEXT

Version 7
01.02.2012

APPENDIX 4: Principal Line Listing Section: Clinical trial, spontaneous, literature and regulatory reports

PRIMARY SYSTEM ORGAN CLASS

CASE COMMENT NUMBER	COUNTRY / SOURCE *	SEX / AGE	DESCRIPTION OF REACTION (MedDRA PT)	DATE OF ONSET OF REACTION (or time to onset)	DATES OF TREATMENT (or duration)	DAILY DOSE	FORM / ROUTE	OUTCOME	
**Gastrointestinal disorders**									
[redacted]		FEMALE	Oral pain (Oral pain)	23-Sep-2010	1.25% Fluoride Gel	NI/NI/	DENTAL GEL / Oral	Unknown	B

1 Oral pain (Oral pain)
2 Oral discomfort (Burning mouth)
3 Oral mucosal exfoliation (Peeling mouth)
4 Oral mucosal erythema (Oral redness)
5 Application site cold feeling (Application site cold feeling)

[redacted]			Tooth discoloration (Tooth discolouration)		1.25% Fluoride Gel	NI/NI/	DENTAL GEL / Oral	Unknown	B

1 Tooth discolouration (Tooth discoloration)

[redacted]		MALE / 11 years	Palatal edema (Palatal oedema)	??-???-2009	1.25% Fluoride Gel	NI/NI/	DENTAL GEL / Oral	Recovered / Resolved

1 Palatal oedema (Palatal edema)
2 Cough (Coughing)
3 Nausea (Nausea)
4 Dry mouth (Dry mouth)

PRIMARY SYSTEM ORGAN CLASS

CASE COMMENT NUMBER	COUNTRY / SOURCE *	SEX / AGE	DESCRIPTION OF REACTION (MedDRA PT)	DATE OF ONSET OF REACTION (or time to onset)	DATES OF TREATMENT (or duration)	DAILY DOSE	FORM / ROUTE	OUTCOME
[redacted]		MALE / 7 years	Yellow teeth (Tooth discolouration)		1.25% Fluoride Gel			Unknown
						NI/QW/	DENTAL GEL / Oral	
	1	Tooth discolouration (Teeth yellow)						
[redacted]		FEMALE / 25 years	Stomatitis (Stomatitis)	??-May-2010	1.25% Fluoride Gel			Recovered / Resolved
					??-May-2010 to ?? -May-2010	[Recommended dose in the package insert]	DENTAL GEL / Oral	
	1	Stomatitis (Stomatitis)						
	2	Oral discomfort (Burning oral sensation)						
	3	Oral pruritus (Itching mouth)						
	4	Oedema mouth (Oral mucosa swollen)						
	5	Oral mucosal exfoliation (Oral mucosal exfoliation)						
	6	Pain (Pain)						
	7	Hypoaesthesia (Numbness)						
[redacted]		MALE / 75 years	Swollen gingiva (Gingival swelling)	08-Sep-2009	1.25% Fluoride Gel			Recovering / Resolving
						NI/NI/	DENTAL GEL / Oral	
	1	Gingival swelling (Gum swelling)						
	2	Gingival erythema (Redness gum)						
	3	Oedema mouth (Swollen mouth)						
	4	Stomatitis (Stomatitis)						

B

PRIMARY SYSTEM ORGAN CLASS

CASE COMMENT NUMBER	COUNTRY / SOURCE *	SEX / AGE	DESCRIPTION OF REACTION (MedDRA PT)	DATE OF ONSET OF REACTION (or time to onset)	DATES OF TREATMENT (or duration)	DAILY DOSE	FORM / ROUTE	OUTCOME
[redacted]		FEMALE	She got swollen lips (Lip swelling)	??-???-2010	1.25% Fluoride Gel			Recovered / Resolved
					??-???-2001 to ??	1 DF 1 times every 1 Week Oral	DENTAL GEL / Oral	
	1	Lip swelling (Swelling of lips)						
	2	Throat irritation (Throat irritation)						
[redacted]			Vomited (Vomiting)	??-Apr-2011	1.25% Fluoride Gel			Unknown
					13-Apr-2011 to 15-Apr-2011	1 DF QID Oral	DENTAL GEL / Oral	
	1	Vomiting (Vomited)						
	2	Diarrhoea (Diarrhea)						
	3	Abdominal pain upper (Stomach ache)						
	4	Pyrexia (Fever)						
[redacted]		FEMALE	Swallowing difficult (Dysphagia)		1.25% Fluoride Gel			Recovered / Resolved
						NI [bite tray for 5 minutes]/NI/	DENTAL GEL / Oral	
	1	Dysphagia (Swallowing difficult)						
	2	Mastication disorder (Chewing difficulty)						
	3	Mucosal haemorrhage (Mucosa bleeding spot)						
	4	Oedema mouth (Oral mucosa swollen)						
[redacted]		MALE	Swelling of tongue (Swollen tongue)	??-???-2010	1.25% Fluoride Gel			Recovered / Resolved
						NI/NI/	DENTAL GEL / Oral	

B

PRIMARY SYSTEM ORGAN CLASS

CASE COMMENT NUMBER	COUNTRY / SOURCE *	SEX / AGE	DESCRIPTION OF REACTION (MedDRA PT)	DATE OF ONSET OF REACTION (or time to onset)	DATES OF TREATMENT (or duration)	DAILY DOSE	FORM / ROUTE	OUTCOME
	1 Swollen tongue (Swelling of tongue)							
	2 Obstructive airways disorder (Airway obstruction NOS)							
[redacted]			Tooth pain (Toothache)	??-???-2009	1.25% Fluoride Gel			Unknown
					??-???-2009 to ??	NI/1 time every two months/	DENTAL GEL / Oral	
	1 Toothache (Tooth pain)							
[redacted]			tooth discoloration (Tooth discolouration)		1.25% Fluoride Gel			Unknown
					??-Aug-2011 to ??		DENTAL GEL / Oral	
	1 Tooth discolouration (Tooth discoloration)							
[redacted]		FEMALE	Gingival swelling (Gingival swelling)	05-Jan-2012	1.25% Fluoride Gel			Unknown
					05-Jan-2012 to 05-Jan-2012	[A pea sized amount]	TOOTHPASTE / Oral	
	1 Gingival swelling (Gingival swelling)							
	2 Lip pruritus (Lip pruritus)							
	3 Lip swelling (Lip swelling)							
[redacted]		MALE	Oral mucosa bleeding (Mouth haemorrhage)		1.25% Fluoride Gel			Unknown
						NI/NI/	DENTAL GEL / Oral	

B

PRIMARY SYSTEM ORGAN CLASS

CASE COMMENT NUMBER	COUNTRY / SOURCE *	SEX / AGE	DESCRIPTION OF REACTION (MedDRA PT)	DATE OF ONSET OF REACTION (or time to onset)	DATES OF TREATMENT (or duration)	DAILY DOSE	FORM / ROUTE	OUTCOME
	1 Mouth haemorrhage (Oral mucosa bleeding)							
	2 Dry mouth (Mouth dry)							
	3 Lip dry (Dry lips)							
	4 Dry skin (Dry skin)							
[redacted]		MALE	Swollen tongue (Swollen tongue)		1.25% Fluoride Gel			Recovered / Resolved
						1 DF QD Oral	DENTAL GEL / Oral	
	1 Swollen tongue (Swollen tongue)							
	2 Throat irritation (Burning in throat)							
	3 Gingival erythema (Redness gum)							
[redacted]		FEMALE / 13 years	tongue blister (Tongue blistering)	09-Feb-2012	1.25% Fluoride Gel			Unknown
						NI/NI/	DENTAL GEL / Oral	
	1 Tongue blistering (Tongue blistering)							
[redacted]		FEMALE	Teeth yellow (Tooth discolouration)	??-Feb-2012	1.25% Fluoride Gel			Unknown
					??-Feb-2012 to ?? -???-2012	Normal amount/QWK/	DENTAL GEL / Oral	
					??-???-2012 to ??	NI/NI/	DENTAL GEL / Oral	
	1 Tooth discolouration (Teeth yellow)							

PRIMARY SYSTEM ORGAN CLASS

CASE COMMENT NUMBER	COUNTRY / SOURCE *	SEX / AGE	DESCRIPTION OF REACTION (MedDRA PT)	DATE OF ONSET OF REACTION (or time to onset)	DATES OF TREATMENT (or duration)	DAILY DOSE	FORM / ROUTE	OUTCOME
[redacted]		MALE	teeth yellow (Tooth discolouration)		1.25% Fluoride Gel			Unknown
						NI/ Once/	DENTAL GEL / Oral	
1	Tooth discolouration (Teeth yellow)							
[redacted]		FEMALE	teeth yellow (Tooth discolouration)		1.25% Fluoride Gel			Unknown
						NI/ Once/	DENTAL GEL / Oral	
1	Tooth discolouration (Teeth yellow)							
[redacted]		MALE	Tongue exfoliation (Tongue exfoliation)	??-???-2012	1.25% Fluoride Gel			Not Recovered / Not Resolved
					?? to ??-???-2012	More than 1 cm/ Once a week/	DENTAL GEL / Oral	
1	Tongue exfoliation (Tongue exfoliation)							
2	Oral mucosal exfoliation (Oral mucosal exfoliation)							
3	Oral discomfort (Burning mouth)							
4	Intentional drug misuse (Intentional drug misuse)							
**General disorders and administration site conditions**								
[redacted]		FEMALE	Pain (Pain)		1.25% Fluoride Gel			Unknown
						NI/NI/	DENTAL GEL / Oral	
1	Pain (Pain)							

Immune system disorders

B

PRIMARY SYSTEM ORGAN CLASS

CASE COMMENT NUMBER	COUNTRY / SOURCE *	SEX / AGE	DESCRIPTION OF REACTION (MedDRA PT)	DATE OF ONSET OF REACTION (or time to onset)	DATES OF TREATMENT (or duration)	DAILY DOSE	FORM / ROUTE	OUTCOME
[redacted]		FEMALE / 6 years	Allergic reaction (Hypersensitivity)		1.25% Fluoride Gel<br>??-???-2010 to ?? -Feb-2011	1 DF 1 times every 3 Month Oral	DENTAL GEL / Oral	Recovered / Resolved
			1 Hypersensitivity (Allergic reaction)<br>2 Nasal oedema (Nasal edema)<br>3 Eye swelling (Eye swelling)<br>4 Lip swelling (Swelling of lips)<br>5 Skin irritation (Irritation skin)					
[redacted]		MALE / 58 years	Allergic shock (Anaphylactic shock)	12-Jan-2012	1.25% Fluoride Gel<br>??-Dec-2011 to ??	[About 10 pieces] 3 times per week/	DENTAL GEL / Oral	Recovered / Resolved
			1 Anaphylactic shock (Allergic shock)<br>2 Dyspnoea (Breathing difficult)					
**Injury, poisoning and procedural complications**								
[redacted]		FEMALE / 21 months	Accidental drug intake by child (Accidental drug intake by child)	05-Sep-2009	1.25% Fluoride Gel<br>05-Sep-2009 to 05-Sep-2009	[between 9-11 grams]	DENTAL GEL / Oral	Recovered / Resolved

PRIMARY SYSTEM ORGAN CLASS

CASE COMMENT NUMBER	COUNTRY / SOURCE *	SEX / AGE	DESCRIPTION OF REACTION (MedDRA PT)	DATE OF ONSET OF REACTION (or time to onset)	DATES OF TREATMENT (or duration)	DAILY DOSE	FORM / ROUTE	OUTCOME

1 Accidental drug intake by child (Accidental drug intake by child)
2 Unresponsive to stimuli (Unresponsive to stimuli)
3 Amnestic disorder (Amnestic disorder)
4 Miosis (Pupils constricted)
5 Vomiting (Vomiting)
6 Nausea (Nausea)
7 Somnolence (Drowsiness)
8 Crying (Crying)

CASE COMMENT NUMBER	COUNTRY / SOURCE *	SEX / AGE	DESCRIPTION OF REACTION (MedDRA PT)	DATE OF ONSET OF REACTION (or time to onset)	DATES OF TREATMENT (or duration)	DAILY DOSE	FORM / ROUTE	OUTCOME
[redacted]		FEMALE / 11 years	Accidental drug intake by child (Accidental drug intake by child)	09-Dec-2010	1.25% Fluoride Gel<br>09-Dec-2010 to 09-Dec-2010	[dental splint left in for 1 hour, more than recommended]	DENTAL GEL / Oral	Not Recovered / Not Resolved

B

PRIMARY SYSTEM ORGAN CLASS

CASE COMMENT NUMBER	COUNTRY / SOURCE *	SEX / AGE	DESCRIPTION OF REACTION (MedDRA PT)	DATE OF ONSET OF REACTION (or time to onset)	DATES OF TREATMENT (or duration)	DAILY DOSE	FORM / ROUTE	OUTCOME

1 Accidental drug intake by child (Accidental drug intake by child)
2 Drug administration error (Drug misadministration)
3 Hypersensitivity (Allergic reaction)
4 Intentional drug misuse (Intentional misuse by dose change)
5 Mucosal erosion (Mucosal erosion)
6 Stomatitis (Oral mucosal irritation)
7 Body temperature increased (Temperature elevation)
8 Feeling of body temperature change (Feeling of body temperature change)
9 Ageusia (Loss of taste)
10 Dysphagia (Swallowing difficult)
11 Chills (Shivering)
12 Crying (Crying)

Investigations

CASE COMMENT NUMBER	COUNTRY / SOURCE *	SEX / AGE	DESCRIPTION OF REACTION (MedDRA PT)	DATE OF ONSET OF REACTION (or time to onset)	DATES OF TREATMENT (or duration)	DAILY DOSE	FORM / ROUTE	OUTCOME
[redacted]		MALE / 13 years	Heartbeats increased (Heart rate increased)		1.25% Fluoride Gel	NI/NI/	DENTAL GEL / Oral	Recovered / Resolved

1 Heart rate increased (Heartbeats increased)
2 Somnolence (Sleepiness)

Nervous system disorders

CASE COMMENT NUMBER	COUNTRY / SOURCE *	SEX / AGE	DESCRIPTION OF REACTION (MedDRA PT)	DATE OF ONSET OF REACTION (or time to onset)	DATES OF TREATMENT (or duration)	DAILY DOSE	FORM / ROUTE	OUTCOME
[redacted]		MALE / 64 years	Altered taste (Dysgeusia)	??-???-2010	1.25% Fluoride Gel<br>?? to ??-???-2010	NI/3x per week/	DENTAL GEL / Oral	Recovering / Resolving

B

PRIMARY SYSTEM ORGAN CLASS

CASE COMMENT NUMBER	COUNTRY / SOURCE *	SEX / AGE	DESCRIPTION OF REACTION (MedDRA PT)	DATE OF ONSET OF REACTION (or time to onset)	DATES OF TREATMENT (or duration)	DAILY DOSE	FORM / ROUTE	OUTCOME
			1 Dysgeusia (Taste altered)					
			2 Ageusia (Loss of taste)					
**Psychiatric disorders**								
[redacted]		MALE	intentional misuse (Intentional drug misuse)	24-Mar-2012	1.25% Fluoride Gel	NI/NI/	DENTAL GEL / Oral	Unknown
			1 Intentional drug misuse (Intentional misuse)					
			2 Oral pain (Mouth pain)					
**Respiratory, thoracic and mediastinal disorders**								
[redacted]		FEMALE	Pharyngeal erythema (Pharyngeal erythema)		1.25% Fluoride Gel	NI/NI/	DENTAL GEL / Oral	Not Recovered / Not Resolved
			1 Pharyngeal erythema (Pharyngeal erythema)					
[redacted]		MALE	Medication aspiration (Foreign body aspiration)	30-Jun-2011	1.25% Fluoride Gel	NI [left on 1-2 minutes]/NI	DENTAL GEL / Oral	Recovering / Resolving
			1 Foreign body aspiration (Medication aspiration)					
			2 Throat irritation (Throat burning sensation of)					
[redacted]			throat swelling (Pharyngeal oedema)	02-Mar-2012	1.25% Fluoride Gel<br>02-Mar-2012 to 02-Mar-2012	Regular amount/ One time only/	DENTAL GEL / Oral	Recovered / Resolved

B

Page 10 of 12

PRIMARY SYSTEM ORGAN CLASS

CASE COMMENT NUMBER	COUNTRY / SOURCE *	SEX / AGE	DESCRIPTION OF REACTION (MedDRA PT)	DATE OF ONSET OF REACTION (or time to onset)	DATES OF TREATMENT (or duration)	DAILY DOSE	FORM / ROUTE	OUTCOME
	1 Pharyngeal oedema (Throat swelling)							
	2 Respiratory distress (Respiratory distress)							
[redacted]		FEMALE / 77 years	numb throat (Pharyngeal hypoaesthesia)	11-May-2012	1.25% Fluoride Gel<br>11-May-2012 to 11-May-2012	NI/ Once/	DENTAL GEL / Oral	Recovered / Resolved
	1 Pharyngeal hypoaesthesia (Numbness throat)							
	2 Gingival pain (Irritation gum)							
	3 Glossitis (Tongue irritation)							
	4 Glossodynia (Tongue pain)							
**Skin and subcutaneous tissue disorders**								
[redacted]		FEMALE	After applying elmex gelee on the thumb and on the forefinger it caused a reddening (Skin discolouration)		1.25% Fluoride Gel	NI/NI/	DENTAL GEL / Oral	Recovering / Resolving
	1 Skin discolouration (Skin discoloration)							
	2 Skin burning sensation (Burning sensation skin)							
	3 Pain (Pain)							
	4 Application site vesicles (Application site blister)							
[redacted]		FEMALE	Facial swelling (Swelling face)		1.25% Fluoride Gel	NI/NI/	DENTAL GEL / Oral	Recovered / Resolved
	1 Swelling face (Facial swelling)							

PRIMARY SYSTEM ORGAN CLASS

CASE COMMENT NUMBER	COUNTRY / SOURCE *	SEX / AGE	DESCRIPTION OF REACTION (MedDRA PT)	DATE OF ONSET OF REACTION (or time to onset)	DATES OF TREATMENT (or duration)	DAILY DOSE	FORM / ROUTE	OUTCOME
[redacted]		MALE	Blood blister (Blood blister)	07-Apr-2010	1.25% Fluoride Gel			Recovering / Resolving
					??-???-1991 to ??	1 DF 1 times every 1 Week Oral	DENTAL GEL / Oral	
						1 DF 1 times every 1 Week Oral	DENTAL GEL / Oral	

1 Blood blister (Blood blister)

Total Case Count: 35

APPENDIX 5: Cumulative Summary Tabulation, Adverse Reactions Grouped by SOC and Source (All)

Cumulative Summary Tabulation, Adverse Reactions Grouped by SOC and Source (All)

System Organ Class/	Spontaneous		Regulatory	Trials	Literature		
Preferred Term	Serious	Non-serious	Serious	Serious	Serious	Non-serious	Total
**Eye disorders**							
Eye swelling	0	1	0	0	0	0	1
Miosis	1	0	0	0	0	0	1
Sub-total	1	1	0	0	0	0	2
**Gastrointestinal disorders**							
Abdominal pain upper	0	1	0	0	0	0	1
Diarrhoea	0	1	0	0	0	0	1
Dry mouth	0	2	0	0	0	0	2
Dysphagia	1	1	0	0	0	0	2
Gingival erythema	0	1	1	0	0	0	2
Gingival pain	0	1	0	0	0	0	1
Gingival swelling	0	1	1	0	0	0	2
Glossitis	0	1	0	0	0	0	1
Glossodynia	0	1	0	0	0	0	1
Lip dry	0	1	0	0	0	0	1
Lip pruritus	0	1	0	0	0	0	1
Lip swelling	0	3	0	0	0	0	3
Mouth haemorrhage	0	1	0	0	0	0	1
Nausea	1	1	0	0	0	0	2
Oedema mouth	1	1	1	0	0	0	3
Oral discomfort	1	2	0	0	0	0	3
Oral mucosal erythema	0	1	0	0	0	0	1
Oral mucosal exfoliation	1	2	0	0	0	0	3
Oral pain	0	2	0	0	0	0	2
Oral pruritus	1	0	0	0	0	0	1

Page 1 of 5

System Organ Class/	Spontaneous		Regulatory	Trials	Literature		
Preferred Term	Serious	Non-serious	Serious	Serious	Serious	Non-serious	Total
Palatal oedema	0	1	0	0	0	0	1
Stomatitis	2	0	1	0	0	0	3
Swollen tongue	0	2	0	0	0	0	2
Tongue blistering	0	1	0	0	0	0	1
Tongue exfoliation	0	1	0	0	0	0	1
Tooth discolouration	0	6	0	0	0	0	6
Toothache	0	1	0	0	0	0	1
Vomiting	1	1	0	0	0	0	2
Sub-total	9	38	4	0	0	0	51
**General disorders and administration site conditions**							
Application site cold feeling	0	1	0	0	0	0	1
Application site vesicles	0	1	0	0	0	0	1
Chills	1	0	0	0	0	0	1
Crying	2	0	0	0	0	0	2
Feeling of body temperature change	1	0	0	0	0	0	1
Mucosal erosion	1	0	0	0	0	0	1
Mucosal haemorrhage	0	1	0	0	0	0	1
Pain	1	2	0	0	0	0	3
Pyrexia	0	1	0	0	0	0	1
Sub-total	6	6	0	0	0	0	12
**Immune system disorders**							
Anaphylactic shock	1	0	0	0	0	0	1
Hypersensitivity	1	1	0	0	0	0	2
Sub-total	2	1	0	0	0	0	3

System Organ Class/ Preferred Term	Spontaneous Serious	Spontaneous Non-serious	Regulatory Serious	Trials Serious	Literature Serious	Literature Non-serious	Total
**Injury, poisoning and procedural complications**							
Accidental drug intake by child	2	0	0	0	0	0	2
Drug administration error	1	0	0	0	0	0	1
Sub-total	3	0	0	0	0	0	3
**Investigations**							
Body temperature increased	1	0	0	0	0	0	1
Heart rate increased	0	1	0	0	0	0	1
Sub-total	1	1	0	0	0	0	2
**Musculoskeletal and connective tissue disorders**							
Mastication disorder	0	1	0	0	0	0	1
Sub-total	0	1	0	0	0	0	1
**Nervous system disorders**							
Ageusia	1	1	0	0	0	0	2
Amnestic disorder	1	0	0	0	0	0	1
Dysgeusia	0	1	0	0	0	0	1
Hypoaesthesia	1	0	0	0	0	0	1
Somnolence	1	1	0	0	0	0	2
Unresponsive to stimuli	1	0	0	0	0	0	1
Sub-total	5	3	0	0	0	0	8
**Psychiatric disorders**							
Intentional drug misuse	1	2	0	0	0	0	3

System Organ Class/ Preferred Term	Spontaneous Serious	Spontaneous Non-serious	Regulatory Serious	Trials Serious	Literature Serious	Literature Non-serious	Total
Sub-total	1	2	0	0	0	0	3
**Respiratory, thoracic and mediastinal disorders**							
Cough	0	1	0	0	0	0	1
Dyspnoea	1	0	0	0	0	0	1
Foreign body aspiration	0	1	0	0	0	0	1
Nasal oedema	0	1	0	0	0	0	1
Obstructive airways disorder	0	1	0	0	0	0	1
Pharyngeal erythema	0	1	0	0	0	0	1
Pharyngeal hypoaesthesia	0	1	0	0	0	0	1
Pharyngeal oedema	0	1	0	0	0	0	1
Respiratory distress	0	1	0	0	0	0	1
Throat irritation	0	3	0	0	0	0	3
Sub-total	1	11	0	0	0	0	12
**Skin and subcutaneous tissue disorders**							
Blood blister	0	1	0	0	0	0	1
Dry skin	0	1	0	0	0	0	1
Skin burning sensation	0	1	0	0	0	0	1
Skin discolouration	0	1	0	0	0	0	1
Skin irritation	0	1	0	0	0	0	1
Swelling face	0	1	0	0	0	0	1
Sub-total	0	6	0	0	0	0	6

System Organ Class/ Preferred Term	Spontaneous Serious	Spontaneous Non-serious	Regulatory Serious	Trials Serious	Literature Serious	Literature Non-serious	Total
TOTAL	29	70	4	0	0	0	103

Spontaneous	99	adverse reaction(s)
Regulatory	4	adverse reaction(s)
Clinical Trials	0	adverse reaction(s)
Literature	0	adverse reaction(s)

Total Cases Reported: 35

APPENDIX 6: Cumulative Summary Tabulation of Serious, Unlisted Adverse Reactions Grouped by SOC and Source

Cumulative Summary Tabulation of Serious, Unlisted Adverse Reactions Grouped by SOC and Source

System Organ Class/ Preferred Term	Spontaneous Serious Unlisted	Regulatory Serious Unlisted	Trials Serious Unlisted	Literature Serious Unlisted	Total
**Eye disorders**					
Miosis	1	0	0	0	1
Sub-total	1	0	0	0	1
**Gastrointestinal disorders**					
Dysphagia	1	0	0	0	1
Oedema mouth	1	0	0	0	1
Oral discomfort	1	0	0	0	1
Oral pruritus	1	0	0	0	1
Stomatitis	1	0	0	0	1
Sub-total	5	0	0	0	5
**General disorders and administration site conditions**					
Chills	1	0	0	0	1
Crying	2	0	0	0	2
Feeling of body temperature change	1	0	0	0	1
Pain	1	0	0	0	1
Sub-total	5	0	0	0	5
**Immune system disorders**					
Anaphylactic shock	1	0	0	0	1
Sub-total	1	0	0	0	1
**Injury, poisoning and procedural complications**					
Drug administration error	1	0	0	0	1

System Organ Class/ Preferred Term	Spontaneous Serious Unlisted	Regulatory Serious Unlisted	Trials Serious Unlisted	Literature Serious Unlisted	Total
Sub-total	1	0	0	0	1
**Investigations**					
Body temperature increased	1	0	0	0	1
Sub-total	1	0	0	0	1
**Nervous system disorders**					
Ageusia	1	0	0	0	1
Amnestic disorder	1	0	0	0	1
Hypoaesthesia	1	0	0	0	1
Somnolence	1	0	0	0	1
Unresponsive to stimuli	1	0	0	0	1
Sub-total	5	0	0	0	5
**Psychiatric disorders**					
Intentional drug misuse	1	0	0	0	1
Sub-total	1	0	0	0	1
**Respiratory, thoracic and mediastinal disorders**					
Dyspnoea	1	0	0	0	1
Sub-total	1	0	0	0	1

System Organ Class/ Preferred Term	Spontaneous Serious Unlisted	Regulatory Serious Unlisted	Trials Serious Unlisted	Literature Serious Unlisted	Total
TOTAL	21	0	0	0	21

Spontaneous	21	adverse reaction(s)
Regulatory	0	adverse reaction(s)
Clinical Trials	0	adverse reaction(s)
Literature	0	adverse reaction(s)

Appendix 7: Number of Reports by Term (signs, symptoms and diagnoses) from Spontaneous, Clinical Study, Regulatory and Literature Cases: all Listed Serious Reactions from the Principal Line Listing

System Organ Class/	Spontaneous		Regulatory	Trials	Literature		
Preferred Term	Serious	Non-serious	Serious	Serious	Serious	Non-serious	Total
**Gastrointestinal disorders**							
Gingival erythema	0	0	1	0	0	0	1
Gingival swelling	0	0	1	0	0	0	1
Nausea	1	0	0	0	0	0	1
Oedema mouth	0	0	1	0	0	0	1
Oral mucosal exfoliation	1	0	0	0	0	0	1
Stomatitis	1	0	1	0	0	0	2
Vomiting	1	0	0	0	0	0	1
Sub-total	4	0	4	0	0	0	8
**General disorders and administration site conditions**							
Mucosal erosion	1	0	0	0	0	0	1
Sub-total	1	0	0	0	0	0	1
**Immune system disorders**							
Hypersensitivity	1	0	0	0	0	0	1
Sub-total	1	0	0	0	0	0	1
**Injury, poisoning and procedural complications**							
Accidental drug intake by child	2	0	0	0	0	0	2
Sub-total	2	0	0	0	0	0	2

Page 1 of 2

System Organ Class/ Preferred Term	Spontaneous Serious	Spontaneous Non-serious	Regulatory Serious	Trials Serious	Literature Serious	Literature Non-serious	Total
TOTAL	8	0	4	0	0	0	12

Spontaneous	8	adverse reaction(s)
Regulatory	4	adverse reaction(s)
Clinical Trials	0	adverse reaction(s)
Literature	0	adverse reaction(s)

Total Cases Reported: 5

Appendix 8: Number of Reports by Term (signs, symptoms and diagnoses) from Spontaneous, Clinical Study, Regulatory and Literature Cases: all Unlisted Reactions, Serious and Non Serious, from the Principal Line Listing

System Organ Class/	Spontaneous		Regulatory	Trials	Literature		
Preferred Term	Serious	Non-serious	Serious	Serious	Serious	Non-serious	Total
**Eye disorders**							
Eye swelling	0	1	0	0	0	0	1
Miosis	1	0	0	0	0	0	1
Sub-total	1	1	0	0	0	0	2
**Gastrointestinal disorders**							
Abdominal pain upper	0	1	0	0	0	0	1
Diarrhoea	0	1	0	0	0	0	1
Dysphagia	1	1	0	0	0	0	2
Glossitis	0	1	0	0	0	0	1
Glossodynia	0	1	0	0	0	0	1
Lip pruritus	0	1	0	0	0	0	1
Lip swelling	0	2	0	0	0	0	2
Mouth haemorrhage	0	1	0	0	0	0	1
Oedema mouth	1	0	0	0	0	0	1
Oral discomfort	1	0	0	0	0	0	1
Oral pain	0	1	0	0	0	0	1
Oral pruritus	1	0	0	0	0	0	1
Stomatitis	1	0	0	0	0	0	1
Tongue blistering	0	1	0	0	0	0	1
Tongue exfoliation	0	1	0	0	0	0	1
Tooth discolouration	0	6	0	0	0	0	6
Toothache	0	1	0	0	0	0	1
Sub-total	5	19	0	0	0	0	24

Page 1 of 4

System Organ Class/ Preferred Term	Spontaneous Serious	Spontaneous Non-serious	Regulatory Serious	Trials Serious	Literature Serious	Literature Non-serious	Total
**General disorders and administration site conditions**							
Application site cold feeling	0	1	0	0	0	0	1
Application site vesicles	0	1	0	0	0	0	1
Chills	1	0	0	0	0	0	1
Crying	2	0	0	0	0	0	2
Feeling of body temperature change	1	0	0	0	0	0	1
Mucosal haemorrhage	0	1	0	0	0	0	1
Pain	1	2	0	0	0	0	3
Pyrexia	0	1	0	0	0	0	1
Sub-total	5	6	0	0	0	0	11
**Immune system disorders**							
Anaphylactic shock	1	0	0	0	0	0	1
Sub-total	1	0	0	0	0	0	1
**Injury, poisoning and procedural complications**							
Drug administration error	1	0	0	0	0	0	1
Sub-total	1	0	0	0	0	0	1
**Investigations**							
Body temperature increased	1	0	0	0	0	0	1
Heart rate increased	0	1	0	0	0	0	1
Sub-total	1	1	0	0	0	0	2
**Musculoskeletal and connective tissue disorders**							

System Organ Class/	Spontaneous		Regulatory	Trials	Literature		
Preferred Term	Serious	Non-serious	Serious	Serious	Serious	Non-serious	Total
Mastication disorder	0	1	0	0	0	0	1
Sub-total	0	1	0	0	0	0	1
**Nervous system disorders**							
Ageusia	1	1	0	0	0	0	2
Amnestic disorder	1	0	0	0	0	0	1
Hypoaesthesia	1	0	0	0	0	0	1
Somnolence	1	1	0	0	0	0	2
Unresponsive to stimuli	1	0	0	0	0	0	1
Sub-total	5	2	0	0	0	0	7
**Psychiatric disorders**							
Intentional drug misuse	1	2	0	0	0	0	3
Sub-total	1	2	0	0	0	0	3
**Respiratory, thoracic and mediastinal disorders**							
Cough	0	1	0	0	0	0	1
Dyspnoea	1	0	0	0	0	0	1
Foreign body aspiration	0	1	0	0	0	0	1
Nasal oedema	0	1	0	0	0	0	1
Obstructive airways disorder	0	1	0	0	0	0	1
Pharyngeal erythema	0	1	0	0	0	0	1
Pharyngeal hypoaesthesia	0	1	0	0	0	0	1
Pharyngeal oedema	0	1	0	0	0	0	1
Respiratory distress	0	1	0	0	0	0	1

System Organ Class/	Spontaneous		Regulatory	Trials	Literature		
Preferred Term	Serious	Non-serious	Serious	Serious	Serious	Non-serious	Total
Throat irritation	0	3	0	0	0	0	3
Sub-total	1	11	0	0	0	0	12
**Skin and subcutaneous tissue disorders**							
Blood blister	0	1	0	0	0	0	1
Dry skin	0	1	0	0	0	0	1
Skin burning sensation	0	1	0	0	0	0	1
Skin discolouration	0	1	0	0	0	0	1
Swelling face	0	1	0	0	0	0	1
Sub-total	0	5	0	0	0	0	5
TOTAL	21	48	0	0	0	0	69

Spontaneous	69	adverse reaction(s)
Regulatory	0	adverse reaction(s)
Clinical Trials	0	adverse reaction(s)
Literature	0	adverse reaction(s)

Total Cases Reported: 34

APPENDIX 9 - LITERATURE REFERENCES FOR FLUORIDE

- **1) M.C.M. Wong, J. Clarkson, A.-M. Glenny, E.C.M. Lo, V.C.C. Marinho, B.W.K. Tsangl T. Walsh, and H.V. Worthington**

Cochrane Reviews on the benefits/Risks of fluoride toothpastes. J Dent Res 90(5):573-579, 2011

Summary:

This concise review, based on 77 papers, reporting 83 independent trials, presents two Cochrane Reviews undertaken to determine: (1) the relative effectiveness of fluoride toothpastes of different concentrations in preventing dental caries in children and adolescents; and (2) the relationship between the use of topical fluorides in young children and their risk of developing dental fluorosis. To determine the relative effectiveness of fluoride toothpastes of different concentrations, the authors undertook a network meta-analysis utilizing both direct and indirect comparisons from randomized controlled trials (RCTs). The review examining fluorosis included evidence from experimental and observational studies. The findings of the reviews confirm the benefits of using fluoride toothpaste, when compared with placebo, in preventing caries in children and adolescents, but only significantly for fluoride concentrations of 1000 ppm and above. The relative caries-preventive effects of fluoride toothpastes of different concentrations increase with higher fluoride concentration. However, there is weak, unreliable evidence that starting the use of fluoride toothpaste in children under 12 months of age may be associated with an increased risk of fluorosis. The decision of what fluoride levels to use for children under 6 years should be balanced between the risk of developing dental caries and that of mild fluorosis.

- **2) B. Hernández-Castro, M. Vigna-Pérez, L. Doníz-Padilla, M. D. Ortiz- Pérez, E. Jiménez-Capdeville, R. González-Amaro, L. Baranda**

Effect of fluoride exposure on different immune parameters in humans. Immunopharmacology and Immunotoxicology, 2011; 33(1): 169–177

Summary

T regulatory (Treg) cells play an important role in the modulation of the immune response, and are implicated in the pathogenesis of autoimmune diseases. Many people is exposed to fluoride (F), mainly through drinking water. The aim of this work was to assess the possible effect of F exposure on different immune parameters, mainly Treg cells. We studied 61 subjects from a community of the state of Durango, Mexico, where the population is exposed to F levels over 2.0 ppm in drinking water. The data suggest that F exposure seems to be associated with a diminution in the programmed cell death of most immune cells, but is not related with modifications in other immune parameters. F exposure may be associated with interesting and potentially relevant effects on the immune system.

ANNEX 1: All Spontaneously Reported Non-serious Listed Reactions

PRIMARY SYSTEM ORGAN CLASS

CASE COMMENT NUMBER	COUNTRY / SOURCE *	SEX / AGE	DESCRIPTION OF REACTION (MedDRA PT)	DATE OF ONSET OF REACTION (or time to onset)	DATES OF TREATMENT (or duration)	DAILY DOSE	FORM / ROUTE	OUTCOME
**Gastrointestinal disorders**								
[redacted]	Other Health Professional		Patient got a burning feeling in the oral cavity (Oral discomfort)	03-Nov-2009	1.25% Fluoride Gel<br>03-Nov-2009 to ?? -Nov-2009	NI/NI/	DENTAL GEL / Oral	Unknown
	1 Oral discomfort (Burning oral sensation)							
[redacted]	Other Health Professional	FEMALE / 67 years	Patient got a reddening of her gums (Gingival erythema)	??-May-2009	1.25% Fluoride Gel<br>??-May-2009 to ??	[Applied 1-2 minutes on the teeth]	DENTAL GEL / Oral	Not Recovered / Not Resolved
	1 Gingival erythema (Redness gum)<br>2 Gingival pain (Irritation gum)<br>3 Gingival swelling (Gum swelling)<br>4 Cheilitis (Cheilitis)<br>5 Oral discomfort (Burning lips)<br>6 Lip swelling (Lip swelling)<br>7 Tongue disorder (Tongue redness)<br>8 Glossodynia (Burning tongue)<br>9 Swollen tongue (Swelling of tongue)<br>10 Glossitis (Tongue irritation)							
[redacted]	Other Health Professional	MALE	The patient got an erosion of his oral mucosa. (Oral mucosal exfoliation)		1.25% Fluoride Gel	NI/NI/	DENTAL GEL / Oral	Unknown

B

PRIMARY SYSTEM ORGAN CLASS

CASE COMMENT NUMBER	COUNTRY / SOURCE *	SEX / AGE	DESCRIPTION OF REACTION (MedDRA PT)	DATE OF ONSET OF REACTION (or time to onset)	DATES OF TREATMENT (or duration)	DAILY DOSE	FORM / ROUTE	OUTCOME
1 Oral mucosal exfoliation (Oral mucosal exfoliation)								
[redacted]	Other Health Professional	FEMALE	Oral mucosa blister (Oral mucosal blistering)		13 years	1.25% Fluoride Gel<br>[brushed for 4 minutes]	DENTAL GEL / Oral	Recovered / Resolved
1 Oral mucosal blistering (Oral mucosa blister)<br>2 Tongue blistering (Tongue blistering)<br>3 Oral pain (Mouth pain)<br>4 Stomatitis (Mouth irritation)								
[redacted]	Other Health Professional	FEMALE	Gingival erosion (Gingival erosion)			1.25% Fluoride Gel<br>NI/NI/	DENTAL GEL / Oral	Unknown
1 Gingival erosion (Gingival erosion)<br>2 Gingival pain (Gum pain)								
[redacted]	Other Health Professional	MALE	Oral mucosal exfoliation (Oral mucosal exfoliation)			1.25% Fluoride Gel<br>NI/NI/	DENTAL GEL / Oral	Unknown
1 Oral mucosal exfoliation (Oral mucosal exfoliation)								
[redacted]	Other Health Professional	FEMALE	Oral mucosal exfoliation (Oral mucosal exfoliation)			1.25% Fluoride Gel<br>NI/NI/	DENTAL GEL / Oral	Unknown
1 Oral mucosal exfoliation (Oral mucosal exfoliation)								

B

PRIMARY SYSTEM ORGAN CLASS

CASE COMMENT NUMBER	COUNTRY / SOURCE *	SEX / AGE	DESCRIPTION OF REACTION (MedDRA PT)	DATE OF ONSET OF REACTION (or time to onset)	DATES OF TREATMENT (or duration)	DAILY DOSE	FORM / ROUTE	OUTCOME
[redacted]	ther Health Professional	MALE / 14 years	Oral mucosal irritation (Stomatitis)	17-Sep-2009		1.25% Fluoride Gel<br>NI/NI/	DENTAL GEL / Oral	Recovering / Resolving

1 Stomatitis (Oral mucosal irritation)
2 Oral pain (Oral mucosa pain)
3 Lip swelling (Swelling lips)

CASE COMMENT NUMBER	COUNTRY / SOURCE *	SEX / AGE	DESCRIPTION OF REACTION (MedDRA PT)	DATE OF ONSET OF REACTION (or time to onset)	DATES OF TREATMENT (or duration)	DAILY DOSE	FORM / ROUTE	OUTCOME
[redacted]	ther Health Professional		Oral aphthae (Aphthous stomatitis)			1.25% Fluoride Gel<br>NI/NI/	DENTAL GEL / Oral	Recovered / Resolved

1 Aphthous stomatitis (Oral aphthae)
2 Lip blister (Lip blister)

CASE COMMENT NUMBER	COUNTRY / SOURCE *	SEX / AGE	DESCRIPTION OF REACTION (MedDRA PT)	DATE OF ONSET OF REACTION (or time to onset)	DATES OF TREATMENT (or duration)	DAILY DOSE	FORM / ROUTE	OUTCOME
[redacted]	ther Health Professional	FEMALE / 68 years	Oral ulceration (Mouth ulceration)	??-Sep-2009	15-Sep-2009 to 15-Sep-2009	1.25% Fluoride Gel<br>[amount as prescribed in package insert]	DENTAL GEL / Oral	Recovered / Resolved

1 Mouth ulceration (Oral ulceration)
2 Oral pain (Mouth pain)
3 Gingival blister (Blisters gum)
4 Lip swelling (Lip swelling)
5 Oral discomfort (Burning mouth)
6 Allergy test positive (Allergy test positive)

PRIMARY SYSTEM ORGAN CLASS

CASE COMMENT NUMBER	COUNTRY / SOURCE *	SEX / AGE	DESCRIPTION OF REACTION (MedDRA PT)	DATE OF ONSET OF REACTION (or time to onset)	DATES OF TREATMENT (or duration)	DAILY DOSE	FORM / ROUTE	OUTCOME
[redacted]	Other Health Professional	FEMALE / 74 years	Gingival pain (Gingival pain)	04-Sep-2009	1.25% Fluoride Gel			Recovered / Resolved
					??-Sep-2009 to ?? -Sep-2009	[used dose recommended in package insert for 1 min]	DENTAL GEL / Oral	
1 Gingival pain (Gingival pain)								
[redacted]	Other Health Professional	FEMALE	Blistering of mouth (Oral mucosal blistering)	2 days after first dose	1.25% Fluoride Gel			Unknown
						Used 2 times/NI/	DENTAL GEL / Oral	
1 Oral mucosal blistering (Blistering of mouth)								
[redacted]	[redacted]ther Health Professional	MALE	Blistering of mouth (Oral mucosal blistering)	2 days after first dose	1.25% Fluoride Gel			Unknown
						Used 2 times/NI/	DENTAL GEL / Oral	
1 Oral mucosal blistering (Blistering of mouth)								
[redacted]	Other Health Professional	FEMALE / 30 years	Swelling of tongue (Swollen tongue)		1.25% Fluoride Gel			Recovered / Resolved
					??-???-1996 to ??	1 DF 1 times every 1 Week Oral	DENTAL GEL / Oral	
					?? to ??-???-2009	1 DF 1 times every 1 Week Oral	DENTAL GEL / Oral	
1 Swollen tongue (Swelling of tongue)								
2 Hypoaesthesia oral (Numbness of tongue)								

PRIMARY SYSTEM ORGAN CLASS

CASE COMMENT NUMBER	COUNTRY / SOURCE *	SEX / AGE	DESCRIPTION OF REACTION (MedDRA PT)	DATE OF ONSET OF REACTION (or time to onset)	DATES OF TREATMENT (or duration)	DAILY DOSE	FORM / ROUTE	OUTCOME
[redacted]	ther alth Professional	FEMALE	Mouth pain (Oral pain)	??-Jan-2011	1.25% Fluoride Gel			Recovered / Resolved
					??-???-2010 to ?? -Jan-2011	NI [once a week as recommended, but not on a regular basis]/NI	DENTAL GEL / Oral	
	1 Oral pain (Mouth pain)							
	2 Oral disorder (Oral lesion)							
[redacted]	ther th Professional		Oral mucosa pain (Oral pain)	17-Oct-2011	1.25% Fluoride Gel			Unknown
					16-Oct-2011 to 17-Oct-2011	[applied to a splint and used overnight]	DENTAL GEL / Oral	
	1 Oral pain (Oral mucosa pain)							
	2 Burning sensation mucosal (Burning mucosa)							
[redacted]	Other Health Professional		Dry mouth (Dry mouth)		1.25% Fluoride Gel			Unknown
					??-???-2006 to ??	NI/applied for 1 hour in dentist's office/	DENTAL GEL / Oral	
					??-???-2006 to ??	3 g 1 times every 1 Week Oral	DENTAL GEL / Oral	
	1 Dry mouth (Dry mouth)							
[redacted]	Other alth Professional	MALE / 10 years	Gum ulceration (Gingival ulceration)		1.25% Fluoride Gel			Unknown
							DENTAL GEL / Oral	

B

PRIMARY SYSTEM ORGAN CLASS

CASE COMMENT NUMBER	COUNTRY / SOURCE *	SEX / AGE	DESCRIPTION OF REACTION (MedDRA PT)	DATE OF ONSET OF REACTION (or time to onset)	DATES OF TREATMENT (or duration)	DAILY DOSE	FORM / ROUTE	OUTCOME
	1 Gingival ulceration (Gum ulceration)							
[redacted]	Other Health Professional	FEMALE / 45 years	Gingivitis (Gingivitis)	28-Nov-2011		1.25% Fluoride Gel NI/NI/	DENTAL GEL / Oral	Recovered / Resolved
	1 Gingivitis (Gingivitis)							
	2 Erythema (Redness facial)							
**General disorders and administration site conditions**								
[redacted]	/ Pharmacist	FEMALE	Mucosal swelling (Oedema mucosal)		16-Apr-2011 to 17-Apr-2011	1.25% Fluoride Gel NI/NI/	DENTAL GEL / Oral	Recovering / Resolving
	1 Oedema mucosal (Mucosal swelling)							
**Immune system disorders**								
[redacted]	Other Health Professional		Allergic reaction (Hypersensitivity)			1.25% Fluoride Gel NI/NI/	DENTAL GEL / Oral	Unknown
	1 Hypersensitivity (Allergic reaction)							
	2 Dermatitis (Perioral dermatitis)							
	3 Eczema (Eczema facial)							
**Injury, poisoning and procedural complications**								
[redacted]	[redacted]ther Health Professional		Accidental ingestion (Accidental exposure)			1.25% Fluoride Gel NI/NI/	DENTAL GEL / Oral	Unknown

B

PRIMARY SYSTEM ORGAN CLASS

CASE COMMENT NUMBER	COUNTRY / SOURCE *	SEX / AGE	DESCRIPTION OF REACTION (MedDRA PT)	DATE OF ONSET OF REACTION (or time to onset)	DATES OF TREATMENT (or duration)	DAILY DOSE	FORM / ROUTE	OUTCOME
			1 Accidental exposure (Accidental ingestion)					
			2 No adverse event (No adverse event)					
**Skin and subcutaneous tissue disorders**								
[redacted]	[redacted]ysician	MALE	Contact dermatitis (Dermatitis contact)		1.25% Fluoride Gel	NI [used one to two hours and rinsed]/NI/	DENTAL GEL / Oral	Recovered / Resolved
			1 Dermatitis contact (Contact dermatitis)					
			2 Aphthous stomatitis (Oral aphthae)					
			3 Oral mucosal exfoliation (Oral mucosal exfoliation)					
[redacted]	[redacted]ician	FEMALE	Allergic eczema (Dermatitis allergic)	??-???-2011	1.25% Fluoride Gel<br>??-???-2010 to ?? -???-2011	NI/NI/	DENTAL GEL / Oral	Recovered / Resolved
			1 Dermatitis allergic (Allergic eczema)					
			2 Cheilitis (Inflammation lips)					

Total Case Count: 24

Source should be reporter type of Physician, Pharmacist, or Other Health Professional.

System Organ Class<br>Adverse Reaction Term (MEDDRA PT)	Total
**Gastrointestinal disorders**	
Aphthous stomatitis	2
Cheilitis	2
Dry mouth	1
Gingival blister	1
Gingival erosion	1
Gingival erythema	1
Gingival pain	3
Gingival swelling	1
Gingival ulceration	1
Gingivitis	1
Glossitis	1
Glossodynia	1
Hypoaesthesia oral	1
Lip blister	1
Lip swelling	3
Mouth ulceration	1
Oral discomfort	3
Oral disorder	1
Oral mucosal blistering	3
Oral mucosal exfoliation	4
Oral pain	5

System Organ Class<br>Adverse Reaction Term (MEDDRA PT)	Total
Stomatitis	2
Swollen tongue	2
Tongue blistering	1
Tongue disorder	1
**Sub-total**	**44**
**General disorders and administration site conditions**	
No adverse event	1
Oedema mucosal	1
**Sub-total**	**2**
**Immune system disorders**	
Hypersensitivity	1
**Sub-total**	**1**
**Injury, poisoning and procedural complications**	
Accidental exposure	1
**Sub-total**	**1**
**Investigations**	
Allergy test positive	1
**Sub-total**	**1**
**Nervous system disorders**	
Burning sensation mucosal	1
**Sub-total**	**1**
**Skin and subcutaneous tissue disorders**	

System Organ Class<br>Adverse Reaction Term (MEDDRA PT)	Total
Dermatitis	1
Dermatitis allergic	1
Dermatitis contact	1
Eczema	1
Erythema	1
**Sub-total**	**5**
**Grand Total**	**55**

Number of patient cases that are represented in the tabulated terms = 24

ANNEX 2: All Medically Unconfirmed Reactions

PRIMARY SYSTEM ORGAN CLASS

Gastrointestinal disorders

CASE COMMENT NUMBER	COUNTRY / SOURCE *	SEX / AGE	DESCRIPTION OF REACTION (MedDRA PT)	DATE OF ONSET OF REACTION (or time to onset)	DATES OF TREATMENT (or duration)	DAILY DOSE	FORM / ROUTE	OUTCOME	SERIOUS / LISTED
[redacted]	[redacted]sumer or other non health professional	FEMALE	Lip swelling (Lip swelling)			1.25% Fluoride Gel<br>NI/NI/	DENTAL GEL / Oral	Recovering / Resolving	NSL

1 Lip swelling (Lip swelling)
2 Gingival swelling (Gum swelling)
3 Swollen tongue (Swollen tongue)
4 Hypoaesthesia oral (Hypoaesthesia oral)
5 Chapped lips (Lip rough)
6 Gingival pain (Irritation gum)
7 Glossitis (Tongue irritation)

CASE COMMENT NUMBER	COUNTRY / SOURCE *	SEX / AGE	DESCRIPTION OF REACTION (MedDRA PT)	DATE OF ONSET OF REACTION (or time to onset)	DATES OF TREATMENT (or duration)	DAILY DOSE	FORM / ROUTE	OUTCOME	SERIOUS / LISTED
[redacted]	Consumer or other non health professional	FEMALE	Lip redness (Cheilitis)			1.25% Fluoride Gel<br>1 DF 1 times every 1 Week Oral	DENTAL GEL / Oral	Recovering / Resolving	NSL

1 Cheilitis (Lip redness)
2 Oral mucosal erythema (Oral mucosa redness)
3 Lip exfoliation (Peeling lips)
4 Oral mucosal exfoliation (Oral mucosal exfoliation)
5 Oral discomfort (Burning lips)
6 Oral discomfort (Burning oral sensation)

B

PRIMARY SYSTEM ORGAN CLASS

CASE COMMENT NUMBER	COUNTRY / SOURCE *	SEX / AGE	DESCRIPTION OF REACTION (MedDRA PT)	DATE OF ONSET OF REACTION (or time to onset)	DATES OF TREATMENT (or duration)	DAILY DOSE	FORM / ROUTE	OUTCOME	SERIOUS / LISTED
[redacted]	sumer or other non health professional	FEMALE	Mouth edema (Oedema mouth)	07-Jul-2010	1.25% Fluoride Gel	1 DF 1 times every 1 Week Oral	DENTAL GEL / Oral	Recovering / Resolving	NSL
	1 Oedema mouth (Edema mouth)<br>2 Hypoaesthesia oral (Tongue tip numbness of)<br>3 Glossodynia (Burning tongue)								
[redacted]	sumer or other non health professional	FEMALE	Gum bleeding (Gingival bleeding)		1.25% Fluoride Gel	NI/NI/	DENTAL GEL / Oral	Unknown	NSU
	1 Gingival bleeding (Gum bleeding)<br>2 Oral discomfort (Burning oral sensation)<br>3 Gingival pain (Burning gum)<br>4 Gingival erythema (Redness gum)<br>5 Stomatitis (Mouth irritation)								
[redacted]	sumer or other non health professional	FEMALE	Gingival pain (Gingival pain)	??-Mar-2010	1.25% Fluoride Gel<br>02-Mar-2010 to ?? -Mar-2010	[Local application on 1 tooth for 15 minutes/]	DENTAL GEL / Oral	Unknown	NSL
	1 Gingival pain (Gingival pain)<br>2 Gingival swelling (Gum swelling)								

B

*Source is usually a reporter type of Lawyer, Consumer, or Other but could also be Physician, Pharmacist or Other Health Professional

PRIMARY SYSTEM ORGAN CLASS

CASE COMMENT NUMBER	COUNTRY / SOURCE *	SEX / AGE	DESCRIPTION OF REACTION (MedDRA PT)	DATE OF ONSET OF REACTION (or time to onset)	DATES OF TREATMENT (or duration)	DAILY DOSE	FORM / ROUTE	OUTCOME	SERIOUS / LISTED
[redacted]	[redacted]sumer or other non health professional	MALE / 37 years	Tongue blistering (Tongue blistering)	14-Oct-2009	1.25% Fluoride Gel<br>12-Oct-2009 to ??	NI/NI/	DENTAL GEL / Oral	Unknown	NSL
1 Tongue blistering (Tongue blistering)									
[redacted]	[redacted]onsumer or other non health professional	FEMALE	Makes me feel nauseous (Nausea)		1.25% Fluoride Gel	NI/NI/	DENTAL GEL / Oral	Unknown	NSL
1 Nausea (Nausea)									
[redacted]	[redacted]mer or other non health professional	MALE	My tongue was black (Tongue discolouration)	05-Dec-2010	1.25% Fluoride Gel	NI/NI/	DENTAL GEL / Oral	Unknown	NSU
1 Tongue discolouration (Tongue black)									
[redacted]	Consumer or other non health professional	FEMALE / 51 years	Stomach cramps (Abdominal pain upper)	26-Dec-2010	1.25% Fluoride Gel<br>??-Dec-2010 to ??	1 DF 1 times every 1 Week Oral	DENTAL GEL / Oral	Recovering / Resolving	NSU
1 Abdominal pain upper (Stomach cramps)<br>2 Diarrhoea (Diarrhea)<br>3 Nausea (Nausea)									

B

PRIMARY SYSTEM ORGAN CLASS

CASE COMMENT NUMBER	COUNTRY / SOURCE *	SEX / AGE	DESCRIPTION OF REACTION (MedDRA PT)	DATE OF ONSET OF REACTION (or time to onset)	DATES OF TREATMENT (or duration)	DAILY DOSE	FORM / ROUTE	OUTCOME	SERIOUS / LISTED
[redacted]	Consumer or other non health professional	FEMALE	My lip area becomes numb (Hypoaesthesia oral)			1.25% Fluoride Gel<br>NI/NI/	DENTAL GEL / Oral	Unknown	NSL

1 Hypoaesthesia oral (Numb lips)
2 Glossitis (Tongue irritation)
3 Glossodynia (Sensitive tongue)

CASE COMMENT NUMBER	COUNTRY / SOURCE *	SEX / AGE	DESCRIPTION OF REACTION (MedDRA PT)	DATE OF ONSET OF REACTION (or time to onset)	DATES OF TREATMENT (or duration)	DAILY DOSE	FORM / ROUTE	OUTCOME	SERIOUS / LISTED
[redacted]	[redacted]er or other non health professional	FEMALE / 39 years	Tongue white (Tongue discolouration)		?? to ??-???-2010	1.25% Fluoride Gel<br>NI/NI/	DENTAL GEL / Oral	Recovered / Resolved	NSU

1 Tongue discolouration (Tongue white)
2 Glossodynia (Burning tongue)
3 Stomatitis (Oral mucosal irritation)
4 Oral pain (Oral pain)
5 Tongue disorder (Tongue redness)
6 Oral discomfort (Burning oral sensation)

CASE COMMENT NUMBER	COUNTRY / SOURCE *	SEX / AGE	DESCRIPTION OF REACTION (MedDRA PT)	DATE OF ONSET OF REACTION (or time to onset)	DATES OF TREATMENT (or duration)	DAILY DOSE	FORM / ROUTE	OUTCOME	SERIOUS / LISTED
[redacted]	[redacted]mer or other non health professional	MALE / 11 years	Tooth coloured yellow (Tooth discolouration)	??-???-2011	23-Mar-2011 to 30-Mar-2011	1.25% Fluoride Gel<br>1 DF BID Oral	DENTAL GEL / Oral	Recovering / Resolving	NSU

1 Tooth discolouration (Tooth coloured yellow)
2 Tooth disorder (Dental disorder NOS)

B

*Source is usually a reporter type of Lawyer, Consumer, or Other but could also be Physician, Pharmacist or Other Health Professional

PRIMARY SYSTEM ORGAN CLASS

CASE COMMENT NUMBER	COUNTRY / SOURCE *	SEX / AGE	DESCRIPTION OF REACTION (MedDRA PT)	DATE OF ONSET OF REACTION (or time to onset)	DATES OF TREATMENT (or duration)	DAILY DOSE	FORM / ROUTE	OUTCOME	SERIOUS / LISTED
[redacted]	Consumer or other non health professional	FEMALE	Oral mucosal erythema (Oral mucosal erythema)		1.25% Fluoride Gel<br>?? to 04-Apr-2011	[half a centimeter]/NI	DENTAL GEL / Oral	Recovering / Resolving	NSL
	1 Oral mucosal erythema (Oral mucosal erythema)								
[redacted]	Consumer or other non health professional	FEMALE	Gagging (Retching)		1.25% Fluoride Gel	NI/NI	DENTAL GEL / Oral	Unknown	NSU
	1 Retching (Gagging)								
[redacted]	Consumer or other non health professional	FEMALE / 27 years	Tip of tongue was numb (Hypoaesthesia oral)	21-Apr-2011	1.25% Fluoride Gel<br>??-Mar-2011 to 20-Apr-2011	NI [pea-sized amount on whole head of toothbrush]/NI/	DENTAL GEL / Oral	Recovered / Resolved	NSL
	1 Hypoaesthesia oral (Tongue tip numbness of)<br>2 Glossodynia (Tongue pain)<br>3 Paraesthesia oral (Tingling tongue)								
[redacted]	Consumer or other non health professional	FEMALE	Vomiting (Vomiting)		1.25% Fluoride Gel	NI/NI/	DENTAL GEL / Oral	Unknown	NSL
	1 Vomiting (Vomiting)								

B

*Source is usually a reporter type of Lawyer, Consumer, or Other but could also be Physician, Pharmacist or Other Health Professional

PRIMARY SYSTEM ORGAN CLASS

CASE COMMENT NUMBER	COUNTRY / SOURCE *	SEX / AGE	DESCRIPTION OF REACTION (MedDRA PT)	DATE OF ONSET OF REACTION (or time to onset)	DATES OF TREATMENT (or duration)	DAILY DOSE	FORM / ROUTE	OUTCOME	SERIOUS / LISTED
[redacted]	Consumer or other non health professional		Tooth discoloration (Tooth discolouration)			1.25% Fluoride Gel<br>1 DF 1 times every 1 Week Oral	DENTAL GEL / Oral	Unknown	NSU
	1 Tooth discolouration (Tooth discoloration)								
[redacted]	Consumer or other non health professional	MALE	The whole gums come off (Oral mucosal exfoliation)			1.25% Fluoride Gel<br>NI/NI/	DENTAL GEL / Oral	Unknown	NSL
	1 Oral mucosal exfoliation (Sloughing gums)								
[redacted]	[redacted]mer or other non health professional	FEMALE	Lip swelling (Lip swelling)			1.25% Fluoride Gel<br>NI/NI/	DENTAL GEL / Oral	Unknown	NSL
	1 Lip swelling (Lip swelling)<br>2 Gingival pain (Irritation gum)								
[redacted]	[redacted]mer or other non health professional	MALE	tongue tip numbness (Hypoaesthesia oral)			1.25% Fluoride Gel	DENTAL GEL / Oral	Unknown	NSL
	1 Hypoaesthesia oral (Tongue tip numbness of)<br>2 Paraesthesia oral (Tongue abnormal feeling of)								
[redacted]	[redacted]mer or other non health professional	FEMALE / 7 years	teeth staining (Tooth discolouration)		??-Dec-2010 to ??	1.25% Fluoride Gel<br>[Small quantity]	DENTAL GEL / Oral	Unknown	NSU

*Source is usually a reporter type of Lawyer, Consumer, or Other but could also be Physician, Pharmacist or Other Health Professional

PRIMARY SYSTEM ORGAN CLASS

CASE COMMENT NUMBER	COUNTRY / SOURCE *	SEX / AGE	DESCRIPTION OF REACTION (MedDRA PT)	DATE OF ONSET OF REACTION (or time to onset)	DATES OF TREATMENT (or duration)	DAILY DOSE	FORM / ROUTE	OUTCOME	SERIOUS / LISTED
	1 Tooth discolouration (Teeth staining)								
	2 Drug administration error (Drug administration error)								
[redacted]	Consumer or other non health professional	FEMALE	Teeth staining (Tooth discolouration)		1.25% Fluoride Gel<br>?? to 05-Mar-2012	Regular amount/Once a week/	DENTAL GEL / Oral	Recovered / Resolved	NSU
	1 Tooth discolouration (Teeth staining)								
[redacted]	Consumer or other non health professional	FEMALE / 49 years	Palatal disorder (Palatal disorder)	??-Mar-2012	1.25% Fluoride Gel<br>??-Dec-2011 to ??	A bit/QWK/	DENTAL GEL / Oral	Unknown	NSU
	1 Palatal disorder (Palatal disorder)								
	2 Surgery (Surgery)								
[redacted]	Consumer or other non health professional	MALE	Gagging (Retching)	1 day after first dose	1.25% Fluoride Gel	NI/Every week/	DENTAL GEL / Oral	Unknown	NSU
	1 Retching (Gagging)								
	2 Dysgeusia (Taste alteration)								
[redacted]	Consumer or other non health professional	FEMALE / 77 years	Teeth sensitivity (Sensitivity of teeth)	12-Apr-2012	1.25% Fluoride Gel	NI/NI/	DENTAL GEL / Oral	Unknown	NSL

B

*Source is usually a reporter type of Lawyer, Consumer, or Other but could also be Physician, Pharmacist or Other Health Professional

PRIMARY SYSTEM ORGAN CLASS

CASE COMMENT NUMBER	COUNTRY / SOURCE *	SEX / AGE	DESCRIPTION OF REACTION (MedDRA PT)	DATE OF ONSET OF REACTION (or time to onset)	DATES OF TREATMENT (or duration)	DAILY DOSE	FORM / ROUTE	OUTCOME	SERIOUS / LISTED
	1		Sensitivity of teeth (Sensitivity of teeth)						
	2		Condition aggravated (Condition aggravated)						
	3		Drug ineffective (Lack of drug effect)						
	4		Oral disorder (Oral mucosal disorder)						
[redacted]	Consumer or other non health professional	FEMALE	tongue burning (Glossodynia)		1.25% Fluoride Gel	NI/NI/	DENTAL GEL / Oral	Unknown	NSL
	1		Glossodynia (Burning tongue)						
	2		Tongue dry (Tongue dry)						
[redacted]	[redacted]er or other non health professional	FEMALE	gum disorder (Gingival disorder)	??-Jun-2012	1.25% Fluoride Gel			Unknown	NSU
					08-Jun-2012 to 08-Jun-2012	Amount of toothbrush/Once/	DENTAL GEL / Oral		
					10-Jun-2012 to 10-Jun-2012	Amount of toothbrush/Once/	DENTAL GEL / Oral		
	1		Gingival disorder (Gum disorder)						
	2		Salivary hypersecretion (Saliva increased)						
[redacted]	Consumer or other non health professional	FEMALE	swollen mouth (Oedema mouth)	??-Jul-2012	1.25% Fluoride Gel			Unknown	NSL
					??-Jul-2012 to ??	NI/ Once per week/	DENTAL GEL / Oral		

B

*Source is usually a reporter type of Lawyer, Consumer, or Other but could also be Physician, Pharmacist or Other Health Professional

PRIMARY SYSTEM ORGAN CLASS

CASE COMMENT NUMBER	COUNTRY / SOURCE *	SEX / AGE	DESCRIPTION OF REACTION (MedDRA PT)	DATE OF ONSET OF REACTION (or time to onset)	DATES OF TREATMENT (or duration)	DAILY DOSE	FORM / ROUTE	OUTCOME	SERIOUS / LISTED
	1 Oedema mouth (Swollen mouth)								
	2 Lip swelling (Lip swelling)								
	3 Oral pain (Mouth pain)								
**General disorders and administration site conditions**									
[redacted]	Consumer or other non health professional	MALE	Crying (Crying)			1.25% Fluoride Gel<br>NI/NI/	DENTAL GEL / Oral	Unknown	NSU
	1 Crying (Crying)								
	2 Nausea (Nausea)								
[redacted]	Consumer or other non health professional	FEMALE	Feeling unwell (Malaise)			1.25% Fluoride Gel<br>NI/NI/	DENTAL GEL / Oral	Unknown	NSU
	1 Malaise (Feeling unwell)								
	2 Dry mouth (Dry mouth)								
[redacted]	Consumer or other non health professional	FEMALE	Pain (Pain)			1.25% Fluoride Gel<br>NI [Applied gel to plastic teeth splint for bruxism]/NI/	DENTAL GEL / Oral	Unknown	NSU

B

*Source is usually a reporter type of Lawyer, Consumer, or Other but could also be Physician, Pharmacist or Other Health Professional

PRIMARY SYSTEM ORGAN CLASS

CASE COMMENT NUMBER	COUNTRY / SOURCE *	SEX / AGE	DESCRIPTION OF REACTION (MedDRA PT)	DATE OF ONSET OF REACTION (or time to onset)	DATES OF TREATMENT (or duration)	DAILY DOSE	FORM / ROUTE	OUTCOME	SERIOUS / LISTED
	1 Pain (Pain)								
	2 Tongue exfoliation (Tongue exfoliation)								
	3 Hypophagia (Oral intake reduced)								
	4 Feeling of despair (Feeling of despair)								
	5 Oral mucosal blistering (Oral mucosa blistering)								
[redacted]	mer or other non health professional	MALE / 73 years	Whole head feels warm (Pyrexia)	??-Jan-2011	1.25% Fluoride Gel<br>26-Jan-2011 to 26-Jan-2011	[applying with a cotton stick and not rinsing, in the evening after brushing his teeth]	DENTAL GEL / Oral	Recovering / Resolving	NSU
	1 Pyrexia (Fever)								
	2 Gingival oedema (Edema gum)								
	3 Gingival pain (Gingival pain)								
	4 Oedema mouth (Edema mouth)								
	5 Erythema (Face red)								
	6 Aphthous stomatitis (Oral aphthae)								
[redacted]	sumer or other non health professional	FEMALE	throat lump (Sensation of foreign body)	23-Jun-2012	1.25% Fluoride Gel<br>23-Jun-2012 to 07-Jul-2012	1st week pea sized, 2nd wk 3/4 pea size, 3rd wk 1/2 pea size/ Once per week/	DENTAL GEL / Oral	Recovering / Resolving	NSU

B

*Source is usually a reporter type of Lawyer, Consumer, or Other but could also be Physician, Pharmacist or Other Health Professional

PRIMARY SYSTEM ORGAN CLASS

CASE COMMENT NUMBER	COUNTRY / SOURCE *	SEX / AGE	DESCRIPTION OF REACTION (MedDRA PT)	DATE OF ONSET OF REACTION (or time to onset)	DATES OF TREATMENT (or duration)	DAILY DOSE	FORM / ROUTE	OUTCOME	SERIOUS / LISTED

1 Sensation of foreign body (Lump feeling in throat)
2 Dysgeusia (Taste metallic)
3 Headache (Headache)

Immune system disorders

CASE COMMENT NUMBER	COUNTRY / SOURCE *	SEX / AGE	DESCRIPTION OF REACTION (MedDRA PT)	DATE OF ONSET OF REACTION (or time to onset)	DATES OF TREATMENT (or duration)	DAILY DOSE	FORM / ROUTE	OUTCOME	SERIOUS / LISTED
[redacted]	[redacted]nsumer or other non health professional	FEMALE	After using elmex gelee for the first time I got a severe allergic reaction in my mouth. (Hypersensitivity)			1.25% Fluoride Gel NI/NI/	DENTAL GEL / Oral	Unknown	NSL

1 Hypersensitivity (Allergic reaction)

CASE COMMENT NUMBER	COUNTRY / SOURCE *	SEX / AGE	DESCRIPTION OF REACTION (MedDRA PT)	DATE OF ONSET OF REACTION (or time to onset)	DATES OF TREATMENT (or duration)	DAILY DOSE	FORM / ROUTE	OUTCOME	SERIOUS / LISTED
[redacted]	[redacted]er or other non health professional	FEMALE	Allergic reaction (Hypersensitivity)			1.25% Fluoride Gel NI/NI	DENTAL GEL / Oral	Unknown	NSL

1 Hypersensitivity (Allergic reaction)

CASE COMMENT NUMBER	COUNTRY / SOURCE *	SEX / AGE	DESCRIPTION OF REACTION (MedDRA PT)	DATE OF ONSET OF REACTION (or time to onset)	DATES OF TREATMENT (or duration)	DAILY DOSE	FORM / ROUTE	OUTCOME	SERIOUS / LISTED
[redacted]	[redacted]mer or other non health professional	MALE / 26 months	Allergic reaction (Hypersensitivity)	08-Aug-2011	08-Aug-2011 to 08-Aug-2011	1.25% Fluoride Gel NI/	DENTAL GEL / Oral	Recovered / Resolved	SU

B

*Source is usually a reporter type of Lawyer, Consumer, or Other but could also be Physician, Pharmacist or Other Health Professional

PRIMARY SYSTEM ORGAN CLASS

CASE COMMENT NUMBER	COUNTRY / SOURCE *	SEX / AGE	DESCRIPTION OF REACTION (MedDRA PT)	DATE OF ONSET OF REACTION (or time to onset)	DATES OF TREATMENT (or duration)	DAILY DOSE	FORM / ROUTE	OUTCOME	SERIOUS / LISTED

1 Hypersensitivity (Allergic reaction)
2 Swelling face (Forehead swelling)
3 Cyanosis (Blue lips)
4 Pallor (Pale skin)
5 Accidental drug intake by child (Accidental drug intake by child)
6 Vomiting (Vomited)
7 Fatigue (Tiredness)
8 Erythema (Skin red)

CASE COMMENT NUMBER	COUNTRY / SOURCE *	SEX / AGE	DESCRIPTION OF REACTION (MedDRA PT)	DATE OF ONSET OF REACTION (or time to onset)	DATES OF TREATMENT (or duration)	DAILY DOSE	FORM / ROUTE	OUTCOME	SERIOUS / LISTED
[redacted]	Consumer or other non health professional	FEMALE	Allergic reaction (Hypersensitivity)		1.25% Fluoride Gel<br>25 years	According to prescription/sporadically/	DENTAL GEL / Oral	Recovered / Resolved	NSL

B

1 Hypersensitivity (Allergic reaction)
2 Paraesthesia oral (Tingling gum)
3 Gingival pain (Irritation gum)
4 Gingival swelling (Gum swelling)

Injury, poisoning and procedural complications

CASE COMMENT NUMBER	COUNTRY / SOURCE *	SEX / AGE	DESCRIPTION OF REACTION (MedDRA PT)	DATE OF ONSET OF REACTION (or time to onset)	DATES OF TREATMENT (or duration)	DAILY DOSE	FORM / ROUTE	OUTCOME	SERIOUS / LISTED
[redacted]	Consumer or other non health professional	MALE / 7 years	Fluorosis (Fluorosis)		1.25% Fluoride Gel<br>??-Jan-2011 to ??	1 DF 1 times every 1 Week Oral	DENTAL GEL / Oral	Unknown	NSU

1 Fluorosis (Fluorosis)
2 Tooth discolouration (Tooth discolouration)

*Source is usually a reporter type of Lawyer, Consumer, or Other but could also be Physician, Pharmacist or Other Health Professional

PRIMARY SYSTEM ORGAN CLASS

CASE COMMENT NUMBER	COUNTRY / SOURCE *	SEX / AGE	DESCRIPTION OF REACTION (MedDRA PT)	DATE OF ONSET OF REACTION (or time to onset)	DATES OF TREATMENT (or duration)	DAILY DOSE	FORM / ROUTE	OUTCOME	SERIOUS / LISTED
[redacted]	Consumer or other non health professional	FEMALE	Used during pregnancy (Maternal exposure during pregnancy)		1.25% Fluoride Gel<br>29-Aug-2011 to ??	NI/NI/	DENTAL GEL / Oral	Unknown	NSL

1 Maternal exposure during pregnancy (Drug exposure during pregnancy)

2 No adverse event (No adverse event)

Investigations

CASE COMMENT NUMBER	COUNTRY / SOURCE *	SEX / AGE	DESCRIPTION OF REACTION (MedDRA PT)	DATE OF ONSET OF REACTION (or time to onset)	DATES OF TREATMENT (or duration)	DAILY DOSE	FORM / ROUTE	OUTCOME	SERIOUS / LISTED
[redacted]	Consumer or other non health professional	FEMALE / 53 years	blood pressure increased (Blood pressure increased)	30-Mar-2012	1.25% Fluoride Gel<br>30-Mar-2012 to 30-Mar-2012	1 gram sample/ Once/	DENTAL GEL / Oral	Not Recovered / Not Resolved	NSU

1 Blood pressure increased (Blood pressure increased)

2 Dysphagia (Swallowing difficult)

3 Hypoaesthesia oral (Numbness of tongue)

4 Abdominal pain upper (Stomach ache)

5 Malaise (Feeling unwell)

Nervous system disorders

CASE COMMENT NUMBER	COUNTRY / SOURCE *	SEX / AGE	DESCRIPTION OF REACTION (MedDRA PT)	DATE OF ONSET OF REACTION (or time to onset)	DATES OF TREATMENT (or duration)	DAILY DOSE	FORM / ROUTE	OUTCOME	SERIOUS / LISTED
[redacted]	[redacted]er or other non health professional	FEMALE	Burning mucosa (Burning sensation mucosal)		1.25% Fluoride Gel	NI/NI/	DENTAL GEL / Oral	Unknown	NSL

1 Burning sensation mucosal (Burning mucosa)

CASE COMMENT NUMBER	COUNTRY / SOURCE *	SEX / AGE	DESCRIPTION OF REACTION (MedDRA PT)	DATE OF ONSET OF REACTION (or time to onset)	DATES OF TREATMENT (or duration)	DAILY DOSE	FORM / ROUTE	OUTCOME	SERIOUS / LISTED
[redacted]	[redacted]mer or other non health professional	MALE	Taste alteration (Dysgeusia)	18-Aug-2010	1.25% Fluoride Gel	NI/NI/	DENTAL GEL / Oral	Unknown	NSL

PRIMARY SYSTEM ORGAN CLASS

CASE COMMENT NUMBER	COUNTRY / SOURCE *	SEX / AGE	DESCRIPTION OF REACTION (MedDRA PT)	DATE OF ONSET OF REACTION (or time to onset)	DATES OF TREATMENT (or duration)	DAILY DOSE	FORM / ROUTE	OUTCOME	SERIOUS / LISTED
	1 Dysgeusia (Taste alteration)								
[redacted]	Consumer or other non health professional	FEMALE / 67 years	Tongue movement impaired (Tongue paralysis)	15-Jan-2012	1.25% Fluoride Gel			Not Recovered / Not Resolved	NSU
					?? to 13-Jan-2012	NI/once weekly/	DENTAL GEL / Oral		
					14-Jan-2012 to ??	NI/twice weekly/	DENTAL GEL / Oral		

1 Tongue paralysis (Tongue movement impaired)

2 Glossodynia (Tongue pain)

3 Tongue blistering (Tongue blistering)

4 Insomnia (Sleeplessness)

Psychiatric disorders

CASE COMMENT NUMBER	COUNTRY / SOURCE *	SEX / AGE	DESCRIPTION OF REACTION (MedDRA PT)	DATE OF ONSET OF REACTION (or time to onset)	DATES OF TREATMENT (or duration)	DAILY DOSE	FORM / ROUTE	OUTCOME	SERIOUS / LISTED
[redacted]	Consumer or other non health professional	MALE	Insomnia (Insomnia)	??-Jan-2011	1.25% Fluoride Gel			Not Recovered / Not Resolved	NSU
					??-Jan-2011 to ??	NI/NI/	DENTAL GEL / Oral		

1 Insomnia (Insomnia)

Renal and urinary disorders

CASE COMMENT NUMBER	COUNTRY / SOURCE *	SEX / AGE	DESCRIPTION OF REACTION (MedDRA PT)	DATE OF ONSET OF REACTION (or time to onset)	DATES OF TREATMENT (or duration)	DAILY DOSE	FORM / ROUTE	OUTCOME	SERIOUS / LISTED
[redacted]	Consumer or other non health professional	MALE	My urine foams while urinatinating into a wall urinal (Urine abnormality)		1.25% Fluoride Gel			Unknown	NSU
						NI/NI/	DENTAL GEL / Oral		

1 Urine abnormality (Urine abnormality)

Respiratory, thoracic and mediastinal disorders

B

PRIMARY SYSTEM ORGAN CLASS

CASE COMMENT NUMBER	COUNTRY / SOURCE *	SEX / AGE	DESCRIPTION OF REACTION (MedDRA PT)	DATE OF ONSET OF REACTION (or time to onset)	DATES OF TREATMENT (or duration)	DAILY DOSE	FORM / ROUTE	OUTCOME	SERIOUS / LISTED
[redacted]	Consumer or other non health professional	MALE / 42 years	Trouble breathing/paralized after using elmex gel (Dyspnoea)			1.25% Fluoride Gel<br>NI/NI/	DENTAL GEL / Oral	Recovered / Resolved	NSU

1 Dyspnoea (Difficulty breathing)
2 Peripheral coldness (Coldness of skin)
3 Skin discolouration (Discoloration skin)
4 Thirst (Thirst)
5 Vasospasm (Spasm of vein)

CASE COMMENT NUMBER	COUNTRY / SOURCE *	SEX / AGE	DESCRIPTION OF REACTION (MedDRA PT)	DATE OF ONSET OF REACTION (or time to onset)	DATES OF TREATMENT (or duration)	DAILY DOSE	FORM / ROUTE	OUTCOME	SERIOUS / LISTED
[redacted]	umer or other non health professional	FEMALE	choking (Choking)			1.25% Fluoride Gel<br>NI/NI/	DENTAL GEL / Oral	Unknown	NSU

1 Choking (Choking)
2 Cough (Coughing)
3 Nausea (Nausea)
4 Feeling abnormal (Feeling bad)

CASE COMMENT NUMBER	COUNTRY / SOURCE *	SEX / AGE	DESCRIPTION OF REACTION (MedDRA PT)	DATE OF ONSET OF REACTION (or time to onset)	DATES OF TREATMENT (or duration)	DAILY DOSE	FORM / ROUTE	OUTCOME	SERIOUS / LISTED
[redacted]	umer or other non health professional	MALE	Throat swelling (Pharyngeal oedema)	??-???-2011	?? to 19-Sep-2011	1.25% Fluoride Gel<br>1 DF QD Oral	DENTAL GEL / Oral	Recovering / Resolving	NSU

1 Pharyngeal oedema (Throat swelling)
2 Dysphagia (Swallowing difficult)
3 Dry mouth (Dry mouth)
4 Palatal oedema (Edema uvula)

B

PRIMARY SYSTEM ORGAN CLASS

CASE COMMENT NUMBER	COUNTRY / SOURCE *	SEX / AGE	DESCRIPTION OF REACTION (MedDRA PT)	DATE OF ONSET OF REACTION (or time to onset)	DATES OF TREATMENT (or duration)	DAILY DOSE	FORM / ROUTE	OUTCOME	SERIOUS / LISTED
**Skin and subcutaneous tissue disorders**									
[redacted]	[redacted]umer or other non health professional	FEMALE	Red confluent rash cheeks (Rash erythematous)	14-Sep-2010	1.25% Fluoride Gel<br>14-Sep-2010 to ??	NI/NI/	DENTAL GEL / Oral	Not Recovered / Not Resolved	NSU
	1 Rash erythematous (Red confluent rash cheeks)<br>2 Urticaria (Hives)								
[redacted]	[redacted]nsumer or other non health professional	FEMALE / 38 years	Scar (Scar)		1.25% Fluoride Gel<br>??-???-2006 to ?? -???-2010	1 DF 1 times every 1 Week Oral	DENTAL GEL / Oral	Recovering / Resolving	NSU
	1 Scar (Scar)<br>2 Aphthous stomatitis (Oral aphthae)								
**Surgical and medical procedures**									
[redacted]	[redacted]umer or other non health professional	MALE	Tooth extraction (Tooth extraction)	??-Jan-2011	1.25% Fluoride Gel<br>??-???-2009 to ??	NI/NI/	DENTAL GEL / Oral	Unknown	NSU
	1 Tooth extraction (Tooth extraction)<br>2 Dental caries (Dental caries)<br>3 Drug ineffective (Drug ineffective)								

B

Total Case Count: 51

System Organ Class<br>Adverse Reaction Term (MEDDRA PT)	Serious Unlisted	Serious Listed	Non-Serious Unlisted	Non-Serious Listed
**Cardiac disorders**				
Cyanosis	1	0	0	0
**Sub-total**	**1**	**0**	**0**	**0**
**Gastrointestinal disorders**				
Abdominal pain upper	0	0	2	0
Aphthous stomatitis	0	0	0	2
Chapped lips	0	0	0	1
Cheilitis	0	0	0	1
Dental caries	0	0	1	0
Diarrhoea	0	0	1	0
Dry mouth	0	0	0	2
Dysphagia	0	0	2	0
Gingival bleeding	0	0	1	0
Gingival disorder	0	0	1	0
Gingival erythema	0	0	0	1
Gingival oedema	0	0	0	1
Gingival pain	0	0	0	6
Gingival swelling	0	0	0	3
Glossitis	0	0	0	2
Glossodynia	0	0	0	6
Hypoaesthesia oral	0	0	0	6
Lip exfoliation	0	0	0	1
Lip swelling	0	0	0	3

**System Organ Class** **Adverse Reaction Term (MEDDRA PT)**	**Serious Unlisted**	**Serious Listed**	**Non-Serious Unlisted**	**Non-Serious Listed**
Nausea	0	0	0	4
Oedema mouth	0	0	0	3
Oral discomfort	0	0	0	4
Oral disorder	0	0	1	0
Oral mucosal blistering	0	0	0	1
Oral mucosal erythema	0	0	0	2
Oral mucosal exfoliation	0	0	0	2
Oral pain	0	0	0	2
Palatal disorder	0	0	1	0
Palatal oedema	0	0	0	1
Paraesthesia oral	0	0	3	0
Retching	0	0	2	0
Salivary hypersecretion	0	0	1	0
Sensitivity of teeth	0	0	0	1
Stomatitis	0	0	0	2
Swollen tongue	0	0	0	1
Tongue blistering	0	0	1	1
Tongue discolouration	0	0	2	0
Tongue disorder	0	0	1	0
Tongue dry	0	0	0	1
Tongue exfoliation	0	0	0	1
Tooth discolouration	0	0	5	0
Tooth disorder	0	0	1	0
Vomiting	0	1	0	1

System Organ Class<br>Adverse Reaction Term (MEDDRA PT)	Serious Unlisted	Serious Listed	Non-Serious Unlisted	Non-Serious Listed
**Sub-total**	**0**	**1**	**26**	**62**
**General disorders and administration site conditions**				
Condition aggravated	0	0	1	0
Crying	0	0	1	0
Drug ineffective	0	0	0	2
Fatigue	1	0	0	0
Feeling abnormal	0	0	1	0
Malaise	0	0	2	0
No adverse event	0	0	0	1
Pain	0	0	1	0
Pyrexia	0	0	1	0
Sensation of foreign body	0	0	1	0
Thirst	0	0	1	0
**Sub-total**	**1**	**0**	**9**	**3**
**Immune system disorders**				
Hypersensitivity	0	1	0	3
**Sub-total**	**0**	**1**	**0**	**3**
**Injury, poisoning and procedural complications**				
Accidental drug intake by child	0	1	0	0
Drug administration error	0	0	1	0
Fluorosis	0	0	1	0
Maternal exposure during pregnancy	0	0	0	1
**Sub-total**	**0**	**1**	**2**	**1**
**Investigations**				

System Organ Class<br>Adverse Reaction Term (MEDDRA PT)	Serious Unlisted	Serious Listed	Non-Serious Unlisted	Non-Serious Listed
Blood pressure increased	0	0	1	0
**Sub-total**	**0**	**0**	**1**	**0**
**Metabolism and nutrition disorders**				
Hypophagia	0	0	1	0
**Sub-total**	**0**	**0**	**1**	**0**
**Nervous system disorders**				
Burning sensation mucosal	0	0	0	1
Dysgeusia	0	0	0	3
Headache	0	0	1	0
Tongue paralysis	0	0	1	0
**Sub-total**	**0**	**0**	**2**	**4**
**Psychiatric disorders**				
Feeling of despair	0	0	1	0
Insomnia	0	0	2	0
**Sub-total**	**0**	**0**	**3**	**0**
**Renal and urinary disorders**				
Urine abnormality	0	0	1	0
**Sub-total**	**0**	**0**	**1**	**0**
**Respiratory, thoracic and mediastinal disorders**				
Choking	0	0	1	0
Cough	0	0	1	0
Dyspnoea	0	0	1	0
Pharyngeal oedema	0	0	1	0
**Sub-total**	**0**	**0**	**4**	**0**

System Organ Class<br>Adverse Reaction Term (MEDDRA PT)	Serious Unlisted	Serious Listed	Non-Serious Unlisted	Non-Serious Listed
**Skin and subcutaneous tissue disorders**				
Erythema	1	0	0	1
Rash erythematous	0	0	1	0
Scar	0	0	1	0
Skin discolouration	0	0	1	0
Swelling face	1	0	0	0
Urticaria	0	0	1	0
**Sub-total**	**2**	**0**	**4**	**1**
**Surgical and medical procedures**				
Surgery	0	0	1	0
Tooth extraction	0	0	1	0
**Sub-total**	**0**	**0**	**2**	**0**
**Vascular disorders**				
Pallor	1	0	0	0
Peripheral coldness	0	0	1	0
Vasospasm	0	0	1	0
**Sub-total**	**1**	**0**	**2**	**0**
**Grand Total**	**5**	**3**	**57**	**74**

Number of patient cases that are represented in the tabulated terms = 51
